# Early-diverging fungal phyla: taxonomy, species concept, ecology, distribution, anthropogenic impact, and novel phylogenetic proposals

**DOI:** 10.1007/s13225-021-00480-y

**Published:** 2021-09-29

**Authors:** Kerstin Voigt, Timothy Y. James, Paul M. Kirk, André L. C. M. de A. Santiago, Bruce Waldman, Gareth W. Griffith, Minjie Fu, Renate Radek, Jürgen F. H. Strassert, Christian Wurzbacher, Gustavo Henrique Jerônimo, David R. Simmons, Kensuke Seto, Eleni Gentekaki, Vedprakash G. Hurdeal, Kevin D. Hyde, Thuong T. T. Nguyen, Hyang Burm Lee

**Affiliations:** 1 Jena Microbial Resource Collection, Leibniz Institute for Natural Product Research and Infection Biology, Adolf-Reichwein-Straße 23, 07745 Jena, Germany; 2 Institute of Microbiology, Faculty of Biological Sciences, Friedrich Schiller University Jena, Neugasse 25, 07743 Jena, Germany; 3 Department of Ecology and Evolutionary Biology, University of Michigan, Ann Arbor, MI 48109 USA; 4 Biodiversity Informatics and Spatial Analysis, Jodrell Laboratory, Royal Botanic Gardens Kew, Surrey, TW9 3DS UK; 5 Department of Mycology, Federal University of Pernambuco, Av. Da Engenharia, s/n, Recife, PE 50740-4600 Brazil; 6 School of Biological Sciences, Seoul National University, Seoul, 08826 South Korea; 7 Department of Integrative Biology, Oklahoma State University, Stillwater, OK 74078 USA; 8 Institute of Biological, Environmental, and Rural Sciences (IBERS), Aberystwyth University, Aberystwyth, SY23 3DD Wales UK; 9 Institute of Biology/Zoology, Evolutionary Biology, Freie Universität Berlin, 14195 Berlin, Germany; 10 Leibniz Institute of Freshwater Ecology and Inland Fisheries, Ecosystem Research, 12587 Berlin, Germany; 11 Department of Civil, Geo and Environmental Engineering, Technical University of Munich, Garching, Germany; 12 School of Science, Mae Fah Luang University, Chiang Rai, 57100 Thailand; 13 Center of Excellence in Fungal Research, Mae Fah Luang University, Chiang Rai, 57100 Thailand; 14 Environmental Microbiology Lab, Department of Agricultural Biological Chemistry, College of Agriculture & Life Sciences, Chonnam National University, Gwangju, 61186 South Korea

**Keywords:** Early-diverging fungi, Evolution, Nuclear large subunit (LSU/28S), Nuclear small subunit (SSU/18S), Molecular phylogeny

## Abstract

The increasing number of new fungal species described from all over the world along with the use of genetics to define taxa, has dramatically changed the classification system of early-diverging fungi over the past several decades. The number of phyla established for non-Dikarya fungi has increased from 2 to 17. However, to date, both the classification and phylogeny of the basal fungi are still unresolved. In this article, we review the recent taxonomy of the basal fungi and re-evaluate the relationships among early-diverging lineages of fungal phyla. We also provide information on the ecology and distribution in *Mucoromycota* and highlight the impact of chytrids on amphibian populations. Species concepts in *Chytridiomycota*, *Aphelidiomycota*, *Rozellomycota*, *Neocallimastigomycota* are discussed in this paper. To preserve the current application of the genus *Nephridiophaga* (*Chytridiomycota*: *Nephridiophagales*)*,* a new type species, *Nephridiophaga blattellae*, is proposed.

## Introduction

Fungi are primarily heterotrophic, nutrition-absorptive (osmotrophic) eukaryotes that exist in every ecological niche. They grow within their food, digesting it externally and absorbing nutrients across a semi-rigid chitinous cell wall during key phases of their vegetative life cycle (Voigt 2012a; James and Berbee 2012; Stajich et al. 2009). A total of 150,246 species of fungi—which match the above-mentioned definition—are currently recognized (Species Fungorum at www.speciesfungorum.org, accessed 19th April, 2021). This is a significant increase on the estimated number (ca. 100,000) reported in 2008 (Kirk et al. 2008). Concurrently, the number of new species annually described in the twenty-first century is rising. Estimations of the extant fungal species range between 1.5 and 3.8 million (Hawksworth 1991, 2001a, b; Hawksworth and Lücking 2017). Assuming no redundancy or error, only 3.8–8.8% of the estimated fungal species were described. This makes a twofold up to 11-fold increase. Molecular operational taxonomic units (MOTUs), in particular, represent a hidden treasure of likely undescribed taxa. Expanding databases to enable the molecular identification of these undescribed taxa should be a priority for fungal taxonomists (Kõljalg et al. 2005). For instance, a study of the basal genus *Mortierella* revealed a large contribution of reference collections to the identification of fungal environmental samples (Nagy et al. 2011).

**Fig. 1:**
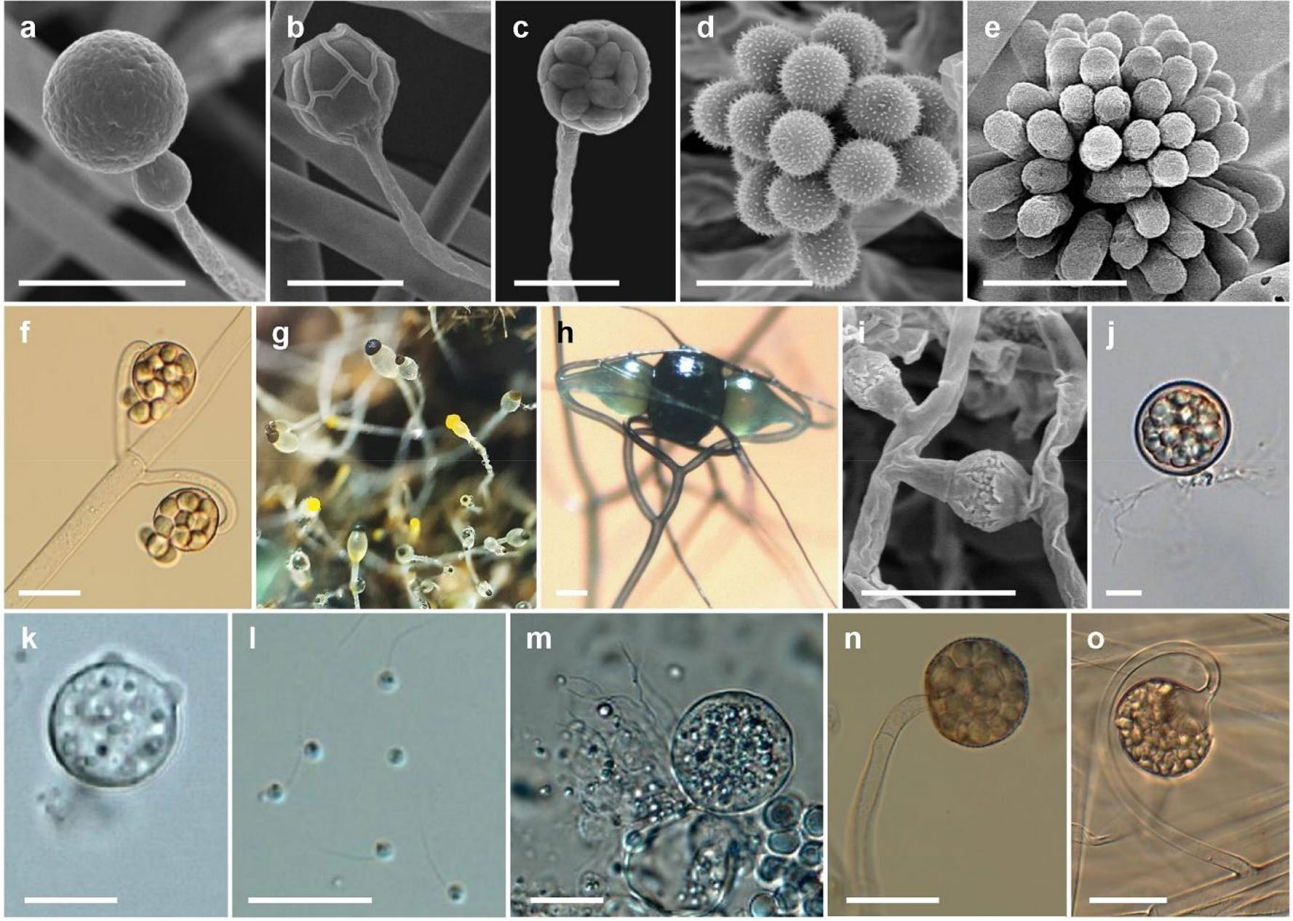
Diversity of basal lineage fungi. **a** *Gongronella koreana* EML-TS2Bp (sporangiophore with sporangium). **b** *Absidia koreana* EML-IFS45-1 (sporangiophore with a sporangium). **c** *Mucor cheongyangensis* CNUFC ICL1 (sporangiophore and mature sporangium). **d** *Cunninghamella elegans* EML-RUS1-1 (vesicle bearing sporangiola). **e** *Syncephalastrum monosporum* EML-BT5-1 (vesicle bearing merosporangiola). **f** *Backusella locustae* CNUFC-SFB2 (multispored sporangiola). **g** *Pilobolus crystallinus* CNUFC-EGF1-4 (yellow and black sporangia at the tips of the sporangiophores on water deer dung). **h** *Syzygites megalocarpus* CNUFC SM01 (zygosporangium with suspensors). **i** *Mucor orantomantidis* CNUFC MID1-1 (zygosporangium with suspensors). **j** *Chytriomyces hyalinus* CNUFC HRW1-3 (mature zoosporangium with branched rhizoids). **k** isolate CNUFC AMS2 (mature thallus with two prominent discharge papillae). **l** isolate CNUFC CHS1-1 (zoospores). **m** isolate CNUFC 19JW3 (mature thallus). **n** isolate CNUFC PS10 (multi-spored sporangiolum). **o** isolate CNUFC IS1 (multi-spored sporangiolum borne on circinate branches). Scale bars = 20 μm, except h, i = 50 μm

Compared to higher fungi (Dikarya), taxonomic and evolutionary studies on the basal fungal lineages are few. There are more than 200 orders of fungi that are classified into a total of 19 fungal phyla (Wijayawardene et al. 2020). Phylogenomic studies are increasingly used to reveal the evolution and phylogeny of fungal taxa. Recently, Galindo et al. (2021) proposed a new phylum, *Sanchytriomycota*, a sister clade to *Blastocladiomycota*, which would increase the number of fungal phyla to 20 of which 17 are basal. Members of basal fungal groups produce both motile and non-motile sporangiospores (Fig. 1). Some species of basal fungi are important in biotechnological areas, such as production of enzymes, lipids and antifungal proteins, and also known as opportunistic pathogens (Walther et al. 2019, 2020). The chytrid fungus *Batrachochytrium dendrobatidis* has been linked to regional and global declines of amphibian populations (Fisher et al. 2009, 2018; Hyde et al. 2018), resulting from disease outbreaks of amphibian chytridiomycosis (Farrer et al. 2011; James et al. 2009; Schloegel et al. 2009). Anaerobic fungi colonize the digestive tracts of herbivorous vertebrates and play a significant role in the breakdown of lignocellulosic feed, providing a source of fermentable sugars for other microbes and the host (Flad et al. 2020). They possess a range of cell wall-degrading enzymes, making them efficient degraders of plant biomass and inexpensive carbohydrates materials (Haitjema et al. 2014). These potent enzymes have received much attention in recent years for their biotechnological and industrial applications.

In this review, we discuss current species concepts, the ecology and distribution of basal lineages of fungi, and propose novel insights into their phylogeny.

## Phylogeny and evolution of early-diverging fungi

Traditional taxonomy splits the fungi into two derived lineages, the *Ascomycota* and the *Basidiomycota*, and two basal lineages, the chytrids or zoosporic (flagellate) fungi (*Chytridiomycota*) and the zygosporic fungi (*Zygomycota*), of which the former phylum as defined by Barr (2001) is aquatic and ancestral to the terrestrial fungi and the latter one among the first terrestrial fungi appearing on Earth (Berbee and Taylor 2001; Liu and Voigt 2010; Mendoza et al. 2014). The morphological diversity of species, which are representative for basal lineage fungi, is illustrated in Fig. 1. The use of gene trees based on DNA sequence data to define taxa has largely accelerated the research on fungal phylogenetics influencing and revolutionizing the systematics of the fungi, especially the classification of the *Zygomycota*, which has changed significantly over the past four decades (Hawksworth et al. 1983, 1995; Kirk et al. 2001, 2008; Voigt and Kirk 2014).

Analysis of large-scale multigene phylogenies and morphological data suggested paraphyletic origins of both basal phyla (James et al. 2006a). The chytrids form zoospores terminally equipped with a single posterior flagellum of the whiplash type (opisthokont) during the vegetative and generative phases of their life cycle. They split into five clades, four of which were given the rank of classes (or even phyla); these are: *Blastocladiomycetes* Doweld (*Blastocladiomycota* T. Y. James: James et al. 2006b); *Chytridiomycetes* Caval.-Sm. (Cavalier-Smith 1998; *Chytridiomycota* Doweld); *Neocallimastigomycetes* M. J. Powell (Hibbett et al. 2007); *Monoblepharidomycetes* J. H. Schaffn. (Schaffner 1909; *Monoblepharidomycota* Doweld). Whilst the *Blastocladiomycetes*, the *Chytridiomycetes* and the *Monoblepharidomycetes* are aerobic-flagellate fungi occurring mainly as saprobes or parasites of plants, animals, protists or algae primarily inhabiting aquatic environments, the *Neocallimastigomycetes* are anaerobic-flagellate fungi that inhabit the digestive systems of herbivorous mammals and reptiles, with the more specialized forms populating the rumen of ruminants (Fliegerova et al. 2012, 2015). Synapomorphic characters are the presence and absence of a prominent basal cell adjacent to the reproductive structure, sexual conjugation via anisogamy, isogamy, oogamy and anaerobic growth for the *Blastocladiomycetes*, the *Chytridiomycetes*, the *Monoblepharidomycetes* and the *Neocallimastigomycetes*, respectively (Voigt 2012b; Shelest and Voigt 2014). The fifth clade of the chytrids consists of the genus *Olpidium*, which comprises unicellular, obligate parasites of plants that reproduce with flagellate, swimming zoospores (Voigt et al. 2013). This genus remains a distinct clade at present, though *Olpidium* was postulated to form a monophyletic group with taxa traditionally classified in the *Zygomycota* (Sekimoto et al. 2011; Chang et al. 2021). Topology tests rejected all alternative trees that constrained the species of *Olpidium* to cluster with other groups of flagellate fungi. The *Zygomycota* is considered to depict the most basal terrestrial phylum to have evolved from flagellate, aquatic ancestors. Molecular phylogenetic analyses revealed dispersal into five subphyla containing one to four orders (Hibbett et al. 2007; Hoffmann et al. 2011).

Based on the potential of all five subphyla to produce zygospores during sexual conjugation of two yoke-shaped gametangia, they are referred to a morphological group named the *Zygomycota* for zygosporic fungi as a whole, which share morphological features but consist of subphyla whose phylogenetic relationships are not completely resolved. Revisions using large-scale multigene phylogenies resulted in conflicting phylogenetic relationships among the basal fungal clades (James et al. 2006a; Liu et al. 2006). The *Zygomycota* group (including *Mucorales*, *Glomerales*, *Entomophthorales* and *Harpellales*) appears monophyletic in a phylogenetic analysis based on the deduced amino acid sequences of the genes encoding RNA polymerase II subunits 1 and 2 (RPB1 and RPB2; Liu et al. 2006), but polyphyletic in a six gene phylogeny using RPB1 and RPB2 in addition to the genes encoding translation elongation factor 1α (TEF), β-tubulin (BTUB) as well as the nuclear small (18S) and the large (28S) subunit ribosomal RNA (rRNA) encoded by the 18S (SSU) and the 28S (LSU) rDNA, respectively (James et al. 2006a). RPB1 and RPB2 were shown to be highly informative and discriminate elegantly at species level, whilst the other marker genes are homoplasious due to convergent or parallel evolution (Schoch et al. 2012).

Nevertheless, the deep-level phylogenetic relationships among the basal lineage fungi remained unsatisfactorily resolved when an oligogenic approach was used for phylogenetic reconstruction. In an endeavour to increase the phylogenetic signal by augmentation of the number of informative characters phylogenomic studies arose, which applied a multitude of orthologous genes and proteins to resolve the deep branches in the fungal tree (Spatafora et al. 2016; Chang et al. 2021).

Members of the phylum *Cryptomycota* M.D.M. Jones & T.A. Richards (Jones et al. 2011a) were proposed to represent evolutionary intermediates between animals and fungi, which cluster at the base of the fungal tree. Contemporarily the new phylum *Rozellomycota* was proposed by James and Berbee (2012) and was validly published by Doweld (2013). The name *Rozellomycota* is used in this review.

Members of the *Rozellomycota* most strikingly lack a chitinous cell wall during food absorption (James and Berbee 2012) and are almost exclusively known from ubiquitous environmental samples by sequencing genes encoding ribosomal RNA (rRNA) (Lara et al. 2010; Jones et al. 2011b). It has been proposed that the *Rozellomycota* may be divergent fungi that evolved from an ancestor with a nearly complete suite of classical fungal-specific characters (James and Berbee 2012). The nuclear genome of *Rozella allomycis* encodes four chitin synthases, including one with a myosin domain, and lacks a large number of genes for primary metabolism (James et al. 2013). *Rozella* is a genus of endoparasites of a range of primarily water mold hosts and a key to a total of 27 species of the genus was published by Letcher and Powell (2018). Molecular phylogenies based on SSU rRNA revealed the existence of a large and widespread group of eukaryotes, branching at the base of the fungal tree (Corsaro et al. 2014). This group, comprising almost exclusively environmental clones, includes the endoparasitic *Rozella* as the unique known representative and two endonuclear rozellids, which have microsporidia-like ultrastructural features and parasitize free-living naked amoebae. Similar to microsporidia, these endoparasites form unflagellated walled spores and grow inside the host cells as unwalled non-phagotrophic meronts and were named *Paramicrosporidium*, appearing to be the morphological missing link between fungi and Microsporidia. Classification system of basal fungi is still controversy. In this paper, we reviewed the current classification of basal fungi in Wijayawardene et al. (2020), and Galindo et al. (2021). Seventeen phyla of basal fungi are shown in Table 1.

**Table 1:** Outline of current classification of basal fungi

Phylum^a^	Class^b^	Order^c^
***Aphelidiomycota*** Tedersoo, Sanchez-Ramirez, Kõljalg, Bahram, M. Döring, Schigel, T.W. May, M. Ryberg & Abarenkov	*Aphelidiomycetes* Tedersoo, Koljalg, Bahram, Doring, Schigel, T. May, Sanchez-Ramirez, M. Ryberg & Abarenkov	*Aphelidiales* Tedersoo, Sanchez-Ramirez, Kõljalg, Bahram, M. Döring, Schigel, T.W. May, M. Ryberg & Abarenkov
***Basidiobolomycota*** Doweld	*Basidiobolomycetes* Humber	*Basidiobolales* Jacz. & P.A. Jacz.
***Blastocladiomycota*** T.Y. James	*Blastocladiomycetes* Doweld	*Blastocladiales* H.E. Petersen *Callimastigales* Doweld *Catenomycetales* Doweld
	*Physodermatomycetes* Tedersoo, Sanchez-Ramirez, Kõljalg, Bahram, M. Döring, Schigel, T.W. May, M. Ryberg & Abarenkov	*Physodermatales* Caval.-Sm.
***Calcarisporiellomycota*** Tedersoo, Sanchez-Ramirez, Kõljalg, Bahram, M. Döring, Schigel, T.W. May, M. Ryberg & Abarenkov	*Calcarisporiellomycetes* Tedersoo, Sanchez-Ramirez, Kõljalg, Bahram, M. Döring, Schigel, T.W. May, M. Ryberg & Abarenkov	*Calcarisporiellales* Tedersoo, Sanchez-Ramirez, Kõljalg, Bahram, M. Döring, Schigel, T.W. May, M. Ryberg & Abarenkov
***Caulochytriomycota*** ^d^Doweld	*Caulochytriomycetes* Doweld	*Caulochytriales* Doweld
***Chytridiomycota*** Doweld	*Chytridiomycetes* Caval.-Sm.	*Chytridiales* Cohn *Nephridiophagales* Doweld *Polyphagales* Doweld *Saccopodiales* Doweld
	*Cladochytriomycetes* Tedersoo, Koljalg, Bahram, Doring, Schigel, T. May, Sanchez-Ramirez, M. Ryberg & Abarenkov	*Cladochytriales* Mozl.-Standr.
	*Lobulomycetes* Tedersoo, Koljalg, Bahram, Doring, Schigel, T. May, Sanchez-Ramirez, M. Ryberg & Abarenkov	*Lobulomycetales* D.R. Simmons
	*Mesochytriomycetes* Tedersoo, Koljalg, Bahram, Doring, Schigel, T. May, Sanchez-Ramirez, M. Ryberg & Abarenkov	*Gromochytriales* Karpov & Aleoshin *Mesochytriales* Doweld
	*Polychytriomycetes* Tedersoo, Koljalg, Bahram, Doring, Schigel, T. May, Sanchez-Ramirez, M. Ryberg & Abarenkov	*Polychytriales* Longcore & D.R. Simmons
	*Rhizophydiomycetes* Tedersoo, Koljalg, Bahram, Doring, Schigel, T. May, Sanchez-Ramirez, M. Ryberg & Abarenkov	*Rhizophydiales* Letcher
	*Rhizophlyctidomycetes* Tedersoo, Koljalg, Bahram, Doring, Schigel, T. May, Sanchez-Ramirez, M. Ryberg & Abarenkov	*Rhizophlyctidales* Letcher
	*Spizellomycetes* Tedersoo, Koljalg, Bahram, Doring, Schigel, T. May, Sanchez-Ramirez, M. Ryberg & Abarenkov	*Spizellomycetales* D.J.S. Barr
	*Synchytriomycetes* Tedersoo, Koljalg, Bahram, Doring, Schigel, T. May, Sanchez-Ramirez, M. Ryberg & Abarenkov	*Synchytriales* Doweld
***Entomophthoromycota*** Humber	*Entomophthoromycetes* Humber	*Entomophthorales* G. Winter
	*Neozygitomycetes* Humber	*Neozygitales* Humber
***Glomeromycota*** C. Walker & A. Schüßler	*Archaeosporomycetes* Sieverd., G.A. Silva, B.T. Goto & Oehl	*Archaeosporales* C. Walker & A. Schüßler
	*Glomeromycetes* Caval.-Sm.	*Diversisporales* C. Walker & A. Schüßler *Glomerales* J.B. Morton & Benny
	*Paraglomeromycetes* Oehl, G.A. Silva, B.T. Goto & Sieverd	*Paraglomerales* C. Walker & A. Schüßler
***Kickxellomycota*** Tedersoo, Koljalg, Bahram, Doring, Schigel, T. May, Sanchez-Ramirez, M. Ryberg & Abarenkov	*Asellariomycetes* Tedersoo, Koljalg, Bahram, Doring, Schigel, T. May, Sanchez-Ramirez, M. Ryberg & Abarenkov	*Asellarialles* Manier ex Manier & Lichtw.
	*Barbatosporomycetes* Tedersoo, Koljalg, Bahram, Doring, Schigel, T. May, Sanchez-Ramirez, M. Ryberg & Abarenkov	*Barbatosporales* Doweld
	*Dimargaritomycetes* Tedersoo, Koljalg, Bahram, Doring, Schigel, T. May, Sanchez-Ramirez, M. Ryberg & Abarenkov	*Dimargaritales* R.K. Benj.
	*Harpellomycetes* Tedersoo, Koljalg, Bahram, Doring, Schigel, T. May, Sanchez-Ramirez, M. Ryberg & Abarenkov	*Harpellales* Lichtw. & Manier
	*Kickxellomycetes* Tedersoo, Sanchez-Ramirez, Kõljalg, Bahram, M. Döring, Schigel, T.W. May, M. Ryberg & Abarenkov	*Kickxellales* Kreisel ex R.K. Benj.
	*Ramicandelaberomycetes* Tedersoo, Koljalg, Bahram, Doring, Schigel, T. May, Sanchez-Ramirez, M. Ryberg & Abarenkov	*Ramicandelaberales* Doweld
***Monoblepharomycota*** Doweld	*Hyaloraphidiomycetes* Doweld	*Hyaloraphidiales* Doweld
	*Monoblepharidomycetes* J.H. Schaffn	*Monoblepharidales* Sparrow
***Mortierellomycota*** Tedersoo, Sanchez-Ramirez, Kõljalg, Bahram, M. Döring, Schigel, T.W. May, M. Ryberg & Abarenkov	*Mortierellomycetes* Doweld	*Mortierellales* Caval.-Sm.
***Mucoromycota*** Doweld	*Endogonomycetes* Doweld	*Endogonales* Jacz. & P.A. Jacz.
	*Mucoromycetes* Doweld	*Mucorales* Fr.
	*Umbelopsidomycetes* Tedersoo, Sanchez-Ramirez, Kõljalg, Bahram, M. Döring, Schigel, T.W. May, M. Ryberg & Abarenkov	*Umbelopsidales* Spatafora, Stajich & Bonito
***Neocallimastigomycota*** M.J. Powell	*Neocallimastigomycetes* M.J. Powell	*Neocallimastigales* J.L. Li, I.B. Heath & L. Packer
***Olpidiomycota*** Doweld	*Olpidiomycetes* Doweld	*Olpidiales* Caval.-Sm.
***Rozellomycota*** Doweld	*Rudimicrosporea* Sprague	*Metchnikovellida* Vivier
	*Microsporidea* Corliss & Levine	*Amblyosporida* Weiser *Neopereziida* Voronin *Ovavesiculida* Sprague, Becnel & Hazard *Glugeida* Gurley *Nosematida* Labbe
***Zoopagomycota*** Gryganskyi, M.E. Sm., Spatafora & Stajich	*Zoopagomycetes* Doweld	*Zoopagales* Bessey ex R.K. Benj.
***Sanchytriomycota*** Galindo et al. 2021	*Sanchytriomycetes* Tedersoo, Sanchez-Ramirez, Kõljalg, Bahram, M. Döring, Schigel, T.W. May, M. Ryberg & Abarenkov	*Sanchytriales* Tedersoo, Sanchez-Ramirez, Kõljalg, Bahram, M. Döring, Schigel, T.W. May, M. Ryberg & Abarenkov

^a^, ^b^, ^c^ The taxa were adopted from the lists in recently published articles (Wijayawardene et al. 2020 and Galindo et al. 2021). ^d^ Doweld (2014) introduced a phylum, *Caulochytriomycota* based on the morphology and habitat of *Caulochytrium* Voos & L.S. Olive. In 2018, Ahrendt et al. sequenced single-cell genomes for one species of *C. protostelioides* and showed that the species was grouped within the *Chytridiales*. However additional analyses of genome sequences are needed to construct clearer relationship between *C. protostelioides* and related chytrids

**Fig. 2:**
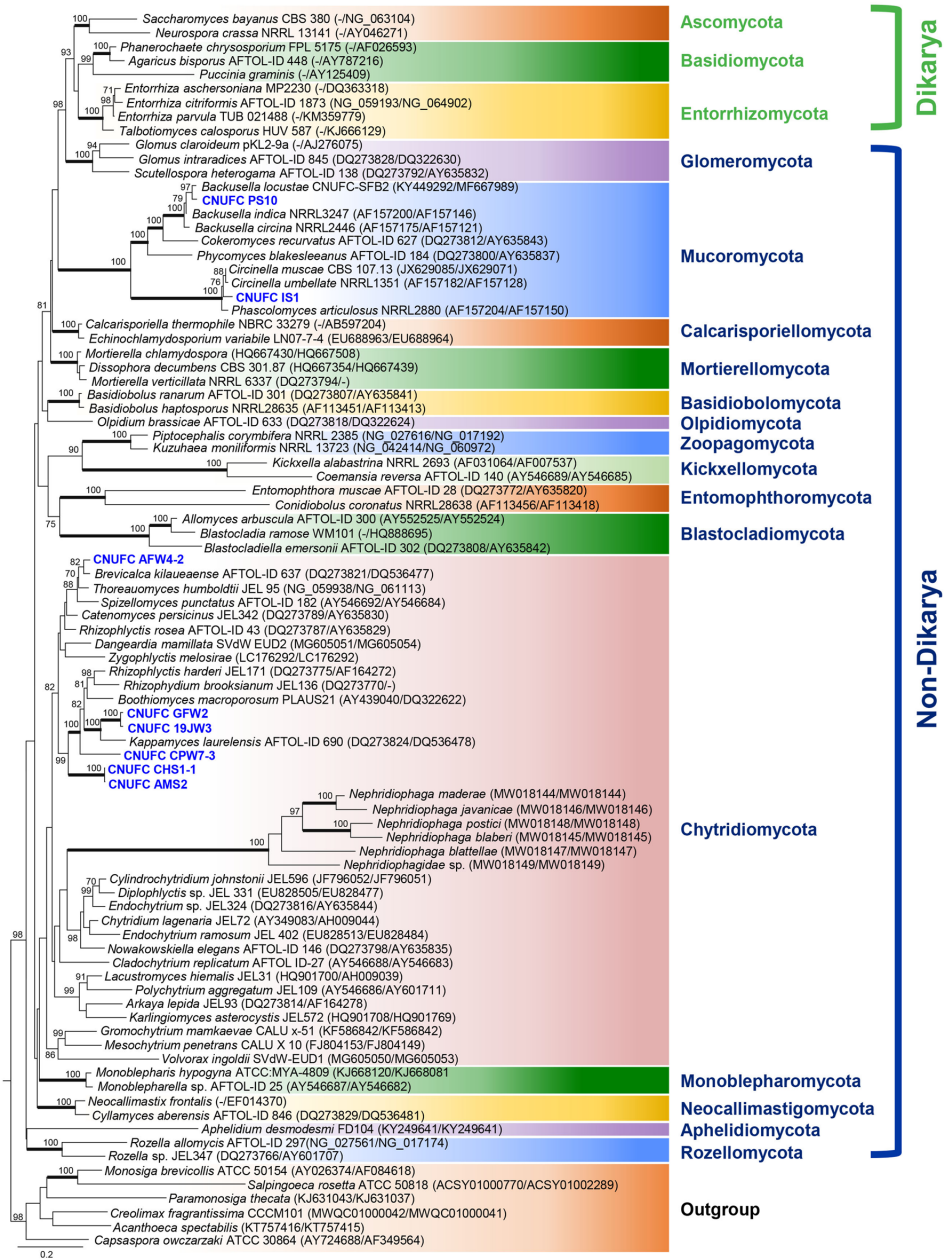
Phylogram generated from Maximum Likelihood analysis based on LSU and SSU combined sequence data showing evolutionary relationships of basal fungal phyla and relatives. The tree was inferred using 89 taxa and 3,790 aligned nucleotide sites. Support in nodes is indicated above or below branches and is represented by bootstrap values (ML analysis) of 70% and higher. Full-supported branches (100% BS) are highlighted by thickened branches. Analysis performed using RAxML-HPC2 on the CIPRES Science Gateway server with 1,000 bootstrap replicates and the GTRGAMMA model of nucleotide substitution. The tree includes six outgroup taxa including *Acanthoeca spectabilis*, *Capsaspora owczarzaki*, *Creolimax fragrantissima*, *Monosiga brevicollis*, *Paramonosiga thecata* and *Salpingoeca rosetta*. Strains in bold blue refer to taxa not designated yet to a specific group. The corresponding sequences were generated during this study

The basal lineages of the fungi appear in a multitude of paraphyletic relationships to the *Dikarya*, encompassing *Ascomycota*, *Basidiomycota*, and *Entorrhizomycota* (Fig. 2). Based on current tree inferences from a concatenated alignment of SSU and LSU rRNA genes of nine nephridiophagid species, the order *Nephridiophagales* has clearly been shown to be affiliated to the *Chytridiomycota* (Strassert et al. 2021). This relationship is confirmed in our phylogenetic analysis (Fig. 2). The analysis performed on the basis of the LSU and SSU combined sequence data (Fig. 2) showed that CNUFC CHS1-1, CNUFC AMS2, CNUFC CPW7-3, CNUFC GFW2, CNUFC 19JW3, and CNUFC AFW4-2 appear to be more related to the phylum *Chytridiomycota*; whereas CNUFC PS10 and CNUFC IS1 grouped within the *Mucorales* (*Mucoromycotina*, *Mucoromycota*). However, they form distinct separate lineages, which may present them as interesting new taxa.

## Species concepts

### Species concepts in *Chytridiomycota*

#### Overview of the problems and methods

In the last 20 years our understanding of the phylogenetic relationships and the taxonomy of the zoosporic true fungi has been completely revised. Phylogenetic studies upended the taxonomy of *Chytridiomycota* by showing it was not a natural group, but rather a paraphyletic grade of lineages retaining motility and diverging long before terrestrial fungi diversified (Letcher et al. 2006; James et al. 2006b; Sekimoto et al. 2011). Robust inference of monophyletic groups was made possible by the use of phylogenies based on DNA sequence data. These data helped to explain the incredible diversity of zoospore ultrastructure and showed that ultrastructural characters of the zoospore could be used to revise the systematics of the group (James et al. 2000; Letcher et al. 2005, 2008a, b; Mozley-Standridge et al. 2009; Simmons et al. 2009). Currently a dual approach combining both DNA sequence and ultrastructural data is used for the taxonomy of zoosporic fungi, and the majority of new taxa, from orders down to species are accompanied with both forms of data (Simmons et al. 2009; Karpov et al. 2014a; Longcore et al. 2020; Seto et al. 2020).

In this period of increased appreciation of zoosporic fungal diversity, the number of phyla has increased to 5 or 7 depending on whether *Rozellomycota* and *Aphelidiomycota* are included within fungi (Karpov et al. 2014b; Tedersoo et al. 2018; James et al. 2020), and the number of orders has blossomed to 22. Many of the new orders reflect taxa that are unculturable, the majority of them phycoparasites or mycoparasites (Karpov et al. 2014a, 2016; Seto et al. 2020). The uncultured and unnamed diversity, the so called “dark matter fungi” (Grossart et al. 2016) apparently comprises a large amount of taxonomic diversity of chytrids. Although the deep relationships among the chytrid phyla and orders have yet to be resolved, orders are now used to describe clearly monophyletic groups, whose ultrastructure is relatively consistent. Revisionary work has been conducted on several of the major orders. During these monographic treatments a number of taxonomic questions have arisen. Firstly, how best should families and genera be defined, and specifically what combination of characters should be used? Gross morphological characters of the thallus have been shown to be nearly useless due to homoplasy (James et al. 2000). Ultrastructural characters are less likely to be homoplastic, but a careful analysis of which characters are more consistent has not been accomplished. Moreover, expertise in interpretation of electron microscopy data is waning and the equipment is not fully accessible to all researchers.

A second set of questions arising from the monographic work is how we should define species, and specifically what species concepts should be employed. In general, the mycological community is focused on using an evolutionary species concept that relies on phylogenetic data to delimit species that are not undergoing genetic exchange (Taylor et al. 2000). Most studies of zoosporic fungal diversity use conserved rRNA (nuclear large and small subunits) genes to generate phylogenies. This tool may not be appropriate to split species, and the use of the non-coding rRNA loci, such as the internal transcribed spacer (ITS) could help (Schoch et al. 2012). Does this move to the use of DNA sequences and ultrastructure in systematic taxonomy leave any room for morphology? Specifically, will morphological or even ecological species concepts have any utility when applied to zoosporic fungi at the species level? Systematists struggle with the fact that most of the named species are recorded in papers published before both widespread culturing of taxa and molecular data existed. In the absence of host information, it is often hard to ascribe isolated chytrid taxa with previous names, particularly given the evidence that suggests most morphological characters are poor indicators of phylogenetic relatedness. Some taxa, however, such as *Lobulomyces poculatus* and *Podochytrium dentatum* are highly distinctive, suggesting that with the right lens or mindset the proper characters could be found for morphological species definitions. For pathogenic species, how stable and useful is host range for delimiting taxa? Could recommendations be provided for future research on zoosporic fungal diversity with respect to defining species concepts across labs? In the following sections we summarize the current state of the taxonomy of three groups of chytrids discussing these taxonomic and evolutionary issues in turn. We include a discussion on how to apply names to observations, cultures, and environmental DNA sequences.

#### Species concepts in *Cladochytriales*

The current members of *Cladochytriales* (seven genera) were previously classified within the *Chytridiales* based on morphological characters of the thallus and developmental stages, but were segregated into a new order after phylogenies of SSU and LSU rDNA showed that the genera were not related to *Chytridiales* (Mozley-Standridge et al. 2009). These data corroborated the differences in zoospore ultrastructure found for these two groups (Lucarotti 1981; Barr 1986; Barr et al. 1987). Currently, classical morphological characters such as operculation, septation on rhizomycelial swellings (turbinate cells), apophysis and catenulations are no longer considered reliable because they are shared by independent lineages (James et al. 2000, 2006b; Powell et al. 2018b; Jerônimo et al. 2019).

The ultrastructural characters of the zoospore have proven to be useful and, in some cases, more informative than morphology. However, in *Cladochytriales*, they still present some obstacles for their use. The necessity of an enormous production of zoospores in pure cultures is a limitation for the polycentric species (e.g. *Nowakowskiella* J. Schrot. and *Cladochytrium* Nowak.), which account for the majority of the order and in general do not produce sufficient zoospores in culture medium (Powell et al. 2018b; Jerônimo et al. 2019). In *Cladochytriales*, only six species (*Allochytridium expandens* Salkin, *Allochytridium luteum* Barr, *Catenochytridium hemicysti* J.S. Knox, *Cladochytrium replicatum* Karling, *Nephrochytrium* sp. JEL125 and *Nowakowskiella elegans* (Nowak.) J. Schrot.)*,* have been investigated by transmission electron microscopy. A unifying zoospore ultrastructural character of the group is the presence and configuration of the microtubular root, in which fibrous linkers connect the microtubules (Mozley-Standridge et al. 2009). This feature and the absence of a paracrystalline inclusion in the zoospore distinguish members of the *Cladochytriales* from *Chytridiales*. Mozley-Standridge et al. (2009) hypothesized that variation in configuration of the fenestrated cisterna may be diagnostic at the generic level, and although it appears to be constant, the fenestrae’s morphology and number of tiers of fenestrae may represent character states. Fenestrae in *Allochytridium expandens*, *Cladochytrium replicatum* and *Nowakowskiella elegans* are elongate and single-tiered. In *Catenochytridium hemicysti* they are compact and single-tiered, while in *Allochytridium luteum* they are compact and multi-tiered (Mozley-Standridge et al. 2009). However, additional studies with a broadly generic sampling are warranted before assessing the value of ultrastructure for taxonomic delineation. For some researchers of zoosporic fungi, molecular data, which are universally obtainable from pure isolates, have been used for making taxonomic revisions without zoospore ultrastructure, especially for taxa that produce zoospores rarely or with extreme difficulty. For example, Steiger et al. (2011) removed *Cylindrochytrium jonhstonii* Karling from *Chytridiales* and transferred it to *Cladochytriales*, and Jerônimo et al. (2019) established a new genus (*Karlingiella* G.H. Jerônimo, A.L. Jesus & Pires-Zottar.) without use of ultrastructural characters.

As the majority of the taxa were described exclusively on morphology, morphology still represents an important starting point for species recognition in *Cladochytriales*. An incorrect morphological identification can cause profound impacts on the systematics of the group in question, since it is highly dependent on both a correct type-species recognition and accurate assignment of names. Although many of the classic morphological characters have been shown to be homoplastic, and morphological knowledge and training has gradually dissipated among new researchers, it is essential for complementation of the molecular studies and taxonomic reviews. Powell et al. (2018b) identified a rare chytrid named *Cladochytrium polystomum* (Zopf 1884) in water bodies from North America. This chytrid is an interesting example of how species are delineated in *Cladochytriales*, since Sparrow (1960) and Karling (1977) questioned whether there was a distinction between *C. polystomum* and *C. replicatum*. Powell et al. (2018b) compared thallus morphology and observed numerous distinctions between those two species. In addition, they also discussed the phylogenetic relationship of *C. polystomum* with the type species of the genus (*Cladochytrium tenue*). According to these authors, *C. polystomum* diverges from *C. replicatum* based on the lack of internal proliferation of zoosporangia, morphology of rhizomycelial swellings, and differences in zoospore ultrastructure in addition to molecular evidence. Phylogenetic analysis demonstrated that *C. polystomum* and *C. tenue* are members of *Chytridiales* instead of *Cladochytriales*, however, and thallus development and molecular data supported the distinction of *C. polystomum* from *C. tenue*. Consequently, the new genus *Zopfochytrium* M.J. Powell, Longcore & Letcher was erected to accommodate the new combination, *Z. polystomum* (Powell et al. 2018b). The phylogenetic placement of *C. tenue* within *Chytridiales* brings to light an important debate on *Cladochytriales* systematics, and according to Powell et al. (2018b), the order needs to be reconsidered in the future when zoospore morphology of *C. tenue* is determined.

Although, *Z. polystomum* differs from *C. replicatum* for the reasons described above, Jerônimo et al. (2019) suggested that *C. replicatum* still represents a complex of multiple species according to the phylogenies of partial SSU and LSU regions of rDNA. Despite the amount of study afforded *C. replicatum*, certain aspects of its morphology need additional clarification. The first point is related to the nature of the resting spores, which were not observed in the original strain (Karling 1931). In subsequent observations, Karling described a similar strain, which produced smooth resting spores, even though spines were present on zoosporangia (Karling 1935). Subsequently, Karling (1937) investigated a strain, which produced both smooth and spiny-walled resting spores, and later, he reported that only 10% of the resting spores in another strain were smooth, the great majority being spiny (Karling 1941). Since none of these studies was made from single-spore isolates, it is entirely possible, as suggested by Sparrow (1960), that there are several distinct species involved. One line of evidence that supports this hypothesis, was the description of two similar species, *C. aureum* (Karling 1949) and *C. aurantiacum* (Richards 1956), which were distinguished from *C. replicatum* by production of spines on resting spores, tubes on the zoosporangium and the coloration of turbinate cells. However, these two species were rarely cited in subsequent studies (Czeczuga et al. 2005), while *C. replicatum* is considered one of the most common and widespread chytrids around the world (Hassan and Shoulkamy 1991; Marano et al. 2008; Jerônimo et al. 2015; Davis et al. 2018), suggesting there may be a consistent misidentification of cultures or observations as *C. replicatum*. From the conflicting evidence concerning morphology and phylogeny, it is possible, even likely, that *C. replicatum* is a species complex, however a careful morphological circumscription, based on comparative analysis of the original descriptions, and molecular characterization are essential for species delineation. This taxonomic problem highlights the complexity of modern taxonomy when the history of the discipline relies on characteristics we now know to be subject to great homoplasy and molecular methods cannot provide a solution because of the absence of type material. *Nephridiophagales* was recently found to form a well-supported monophyletic group within the *Chytridiomyota*, possibly as a sister to the *Cladochytriales* (Strassert et al. 2021). However, the assignment to the *Cladochytriales* is uncertain and awaits further investigation. The ultrastructure of nephridiophagid spores as revealed by transmission electron microscopy does not imply any evidence for flagellae and the kinetosome apparatus required for flagellate movement.

#### Species concepts in *Spizellomycetales*

The *Spizellomycetales* was the first order segregated from Sparrow’s (1960) broad concept of *Chytridiales* based on differing ultrastructural characteristics (Barr 1980). Until that time, morphological comparisons in situ were most often used to define familial and generic rankings. However, morphological variation, most prevalently observed once a fungus was in pure culture, made many investigators question the basis on which these taxonomic divisions were being made. In the 1960s and 1970s, Donald J. S. Barr, together with his colleagues, and his students, began to examine the ultrastructural features of the single-celled, posteriorly-flagellated zoospore of these fungi to determine if more constant, synapomorphic characters were to be found in these propagules. The zoospore is explicitly necessary for reproduction and was thus hyopthesised to be more evolutionarily constrained and conservative. Barr and others observed ultrastructural characters that were used as the defining features for the order *Spizellomycetales*. Among the most conspicuous characters that separated the *Spizellomycetales* from the *Chytridiales* that had been studied at that time were (1) ribosomes scattered throughout the cytoplasm, rather than enclosed in a membrane, as in the *Chytridiales*, (2) a non-flagellated centriole generally at an angle to the kinetosome, (3) a proximity of a portion of the nucleus to the posterior of the zoospore near the kinetosome, (4) microtubules emanating, possibly in multiple directions, from a spur on the kinetosome, and (5) multiple lipid globules permeating the cytoplasm.

*Spizellomycetales* originally included four new genera at the time of its description that would be the cornerstones of the new order. These genera were delineated from each other based on variations, or character states, of the features that distinguished the order from the *Chytridiale*s. More specifically, these genera possessed different character states of the kinetosome spurs and the associated microtubules, ranging from a simple spur seemingly connecting the kinetosome and microtubules, to an elongated spur extending over half the length of the zoospore and encasing a subset of microtubules. The order also included previously described genera, e.g. *Rhizophlyctis* (≡ *Karlingia*), *Rozella*, and *Olpidium*, which exhibited a high degree of variation in their character states, indicative of their phylogenetic unrelatedness to the *Spizellomycetale*s, as has now been recapitulated by molecular methods (James et al. 2006a; Sekimoto et al. 2011). When the *Chytridiomycota* was initially investigated by large-scale molecular phylogenies (James et al. 2000, 2006b), the cornerstone genera of the *Spizellomycetales* were upheld as monophyletic, supporting the group’s delineation based on shared zoosporic characteristics.

Additionally, the new genus *Powellomyces* was described in *Spizellomycetale*s while *Entophlyctis*, previously transitioned to *Spizellomycetales*, was reassigned to the *Chytridiales* (Longcore et al. 1995) based on type species host preference. *Powellomyces* differed morphologically from other spizellomycetalean genera in that it developed exogenously, meaning the zoosporangium was produced exterior to the initial encysted zoospore, rather than the zoospore cyst enlarging to produce the zoosporangium, as in endogenous development. Within the last decade, further study of *Powellomyces* has led to the segregation of three additional genera within this morphological group (Simmons and Longcore 2012), now recognized as the *Powellomycetaceae* (Simmons 2011). These genera were recognized for their molecular monophyly from other *Spizellomycetales* taxa, their development method, and intergeneric ultrastructural character states.

Still, morphology, in correlation with phylogenetic species concepts, is necessary for species designations and identifications to be accessible to a wider mycological audience, which is generally limited to analyses by light microscopy. When the *Spizellomycetales* was described, Barr (1980) made this same argument and highlighted the emphasis on species delimitation by the “abundance of characters” represented by morphology, development, and physiology of these fungi in pure culture. The problem was and remains in determining which characters best predict the molecularly-recognized taxa and are thus phylogenetically useful. To that end, Simmons and Longcore (2012) used a principal components analysis of genera and species in the *Powellomycetaceae* to determine the morphological characters that were most useful for taxon description. Though this analysis did indicate useful features, the study was limited in scope, and the method was not completely accurate in species assignment, given intraspecific morphological variation. Presently, phylogenetic species concepts are thus the gold standard for zoosporic fungi species recognition, but it is necessary for specialists in these groups to provide as many lines of evidence as possible when delimiting new taxa.

As in all fungi, new genera of the *Spizellomycetal*es continue to be found by the production of molecular phylogenies, which indicate cryptic lineages of interest within the larger groupings. When zoospores are examined by electron microscopy, these lineages have shown morphological evidence to support their separation from the previously characterized taxon. This has so far proven to be true of both the exogenously-developing (*Powellomycetaceae*) and endogenously-developing (*Spizellomycetaceae*) members of the *Spizellomycetales*. On the other hand, typically the number of strains examined by electron microscopy is not the same as the number investigated using DNA sequencing, and the level of intraspecific (or lab-to-lab) variation in ultrastructure is largely unexplored. After the description of the *Powellomycetaceae*, the remaining *Spizellomycetales* taxa, which undergo endogenous development, remained in the *Spizellomycetaceae*. In the last five years, the *Spizellomycetaceae* has also shown increased zoosporic variability with further sampling, isolation, and reevaluation, providing evidence for four more genera (Letcher and Powell 2018; Powell et al. 2018a). Though the *Powellomycetaceae* remains monophyletic in a large-scale phylogeny of the order (Simmons et al. 2020), there is molecular evidence that the endogenous *Spizellomycetale*s could be split to separate *Brevicalcar* into its own family, though there is no noteworthy morphological feature, by light or electron microscopy, that would be indicative of this distinction. As convergent thallus morphology has done before, convergent ultrastructural evolution can mask these divergent lineages until an adequate technique is able to observe the differences. As electron microscopy once opened the door to better classification methods, molecular phylogenetics and further techniques will undoubtedly continue to question or reaffirm our current classifications.

#### Species concepts in *Zygophlyctidales*

How obligate parasitic chytrids infect specific hosts and expand their host ranges vary depending on species (Sparrow 1960). In traditional chytrid taxonomy, species delineation of chytrids was based primarily on thallus morphological characters, and host specificity was not considered as a taxonomic criterion for species in many cases. For example, *Micromyces zygogonii* was originally described as an endoparasite of *Zygogonium* sp. (*Zygnematophyceae*) by Dangeard (1889), but many researchers found similar chytrids infecting other genera of zygnematophycean algae (*Mougeotia* and *Spirogyra*) and identified them as *M. zygogonii* (Sparrow 1960). Canter (1963) described *Zygorhizidium cystogenum* parasitic on cysts of *Dinobyrion* and *Uroglena* (Chrysophyceae). Although the chytrids on two host algae differ in their rhizoidal system, they were identified as the same species based on similarity of zoosporangial shape and resting spore with unique covering. Exceptionally, in the plant pathogenic genus *Synchytrium*, which includes > 200 species (Karling 1964), host specificity was often used as a criterion for species identification. Because *Synchytrium* spp. have been considered to have a certain host specificity, parasites occurring on different host plants tended to be described as new species. Actually, more than 50 new species were described based primarily on host plants during the decade of 1950s–1960s (Karling 1964).

However, our knowledge of host specificity of parasitic chytrids largely relies on light microscopic observation and identification of each species. Only a few parasitic chytrids were examined for their host specificity under controlled conditions due to difficulty of cultivation of obligate parasites that cannot grow as pure cultures in general. Therefore, there is a possibility that a “species” infecting multiple host species actually represents a species complex of parasites infecting different hosts with similar morphology. Earlier experimental studies on host specificity of algal parasites using dual cultures revealed that parasitic chytrids are highly (genus or species) specific (Canter and Jaworski 1978, 1986; Canter et al. 1992; Doggett and Porter 1995) in most cases, but Gromov et al. (1999) showed that *Rhizophydium algavorum* had quite a broad host range; it could infect 5 genera and 20 species of green algae as well as the xanthophycean algae *Tribonema gayanum*. In *Synchytrium*, host specificities of some species have been examined by inoculation experiments (summarized in Karling 1964). In many cases, *Synchytrium* spp. were revealed to infect several genera or species of plants, but a few species such as *S. macrosporum* (Karling 1964) and *S. fulgens* (Hartmann 1958) had wider host ranges. Karling (1964) noted that many *Synchytrium* spp. need to be re-examined for their morphology, life cycle, and host specificity.

Difficulty of cultivation also hinders the molecular phylogenetic examination of obligately parasitic chytrids. While saprotrophic chytrids have been well studied in the recent taxonomic revision of zoosporic fungi, most known parasitic species remain to be examined with molecular phylogenetic and zoospore ultrastructural analysis. Recent efforts to establish dual cultures of algal parasites and their taxonomic examination revealed unexpected phylogenetic diversity of parasitic chytrids. Some known as well as newly described parasitic species represented new orders in *Chytridiomycota* (Karpov et al. 2014a; Seto et al. 2020). In other cases, new families or genera of parasitic chytrids were described in the well-studied orders: *Collimycetaceae* (Seto and Degawa 2018a), *Dinomycetaceae* (Lepelletier et al. 2014), *Staurastromycetaceae* (Van den Wyngaert et al. 2017) in *Rhizophydiales* and *Pendulichytrium* in *Chytridiales* (Seto and Degawa 2018b).

The *Zygophlyctidales* is one of the recently described orders represented by parasitic taxa (Seto et al. 2020). This order currently includes three species of diatom parasites, *Zygophlyctis asterionellae*, *Zygop*. *melosirae*, and *Zygop*. *planktonica*, which formerly belonged to the genus *Zygorhizidium*. In these, *Zygop*. *asterionellae* infecting *Asterionella formosa* and *Zygop*. *planktonica* infecting *Ulnaria* spp. (formerly *Synedra*, (Williams 2011)) were known as *Zygorhizidium planktonicum*, which has been extensively studied in the context of ecology and evolution of parasitic chytrids (Van Donk and Ringelberg 1983; de Bruin et al. 2004, 2008; Kagami et al. 2007). However, the species concept of *Zygor*. *planktonicum* was controversial. *Zygor*. *planktonicum* was originally described by Canter and Lund (1953) as a chytrid infecting both *Asterionella* and *Ulnaria*. In contrast, Pongratz (1966) suggested that *Zygor*. *planktonicum* on *Asterionella* and *Ulnaria*, respectively, are host specific based on the observation of phytoplankton in the same lake throughout the year and erected *Zygor*. *asterionellae* for the chytrid on *Asterionella*. Later, dual cultures of *Zygor*. *planktonicum* infecting *Asterionella* (Canter and Jaworski 1986) and *Ulnaria* (Canter et al. 1992) were established and cross inoculation experiments were conducted. The results showed that *Zygor*. *planktonicum* on *Asterionella* infects only *Asterionella* and could not infect *Ulnaria* spp., and vice versa. Canter et al. (1992) concluded that these two host-specific chytrids are the single species *Zygor*. *planktonicum*, but proposed formae speciales for two host specific variants. Afterward, the name *Zygor*. *planktonicum* has been used for chytrids on *Asterionella* (Van Donk and Ringelberg 1983; de Bruin et al. 2004, 2008; Kagami et al. 2007) and *Ulnaria* (Doggett and Porter 1995, 1996).

Recently, the species concept of *Zygor*. *planktonicum* was re-examined based on molecular phylogeny as well as host specificity (Seto et al. 2020). Two dual cultures of *Zygor*. *planktonicum* on *Asterionella* and *Ulnaria*, respectively, were revealed to be highly specific to their original host as with earlier studies (Canter and Jaworski 1986; Canter et al. 1992), and they were clearly phylogenetically distinguished. Additionally, the two cultures differed in zoospore ultrastructure, specifically in the contents of a fibrillar vesicle associated with the fenestrated cisterna. Seto et al. (2020) concluded that two host specific variants of *Zygor*. *planktonicum* warranted separation into distinct species (transferred to the genus *Zygophlyctis*), *Zygop*. *asterionellae* on *Asterionella* and *Zygop*. *planktonica* on a species of diatom, *Ulnaria*.

Interestingly, *Zygop*. *planktonica* can infect more than one species of *Ulnaria* but it prefers a single species and infects other species weakly (Canter et al. 1992; Doggett and Porter 1995). A similar phenomenon was observed in *Zygop*. *melosirae* infecting *Aulacoseira* spp. (Seto et al. 2020). Two strains of *Zygop*. *melosirae* were examined, one parasitizing *A. granulata*, and the other on *A. ambigua*. Although the two chytrids were almost identical in rDNA sequences, they could be distinguished based on host preference. Both chytrids could parasitize the two species of *Aulacoseira*, but heavily on the original host species and weakly on the other species. These two chytrids were regarded as intraspecific variants in a single species *Zygop*. *melosirae* based on the identity of rDNA sequences. From these results, zygophlyctidaleean chytrids are specific to a single diatom genus, but each chytrid species includes several intraspecific variants preferring a certain diatom species. Therefore, in *Zygophlyctidales*, host genus could be an important taxonomic criterion for species delineation, and is thus far supported by molecular phylogenetics.

**Fig. 3:**
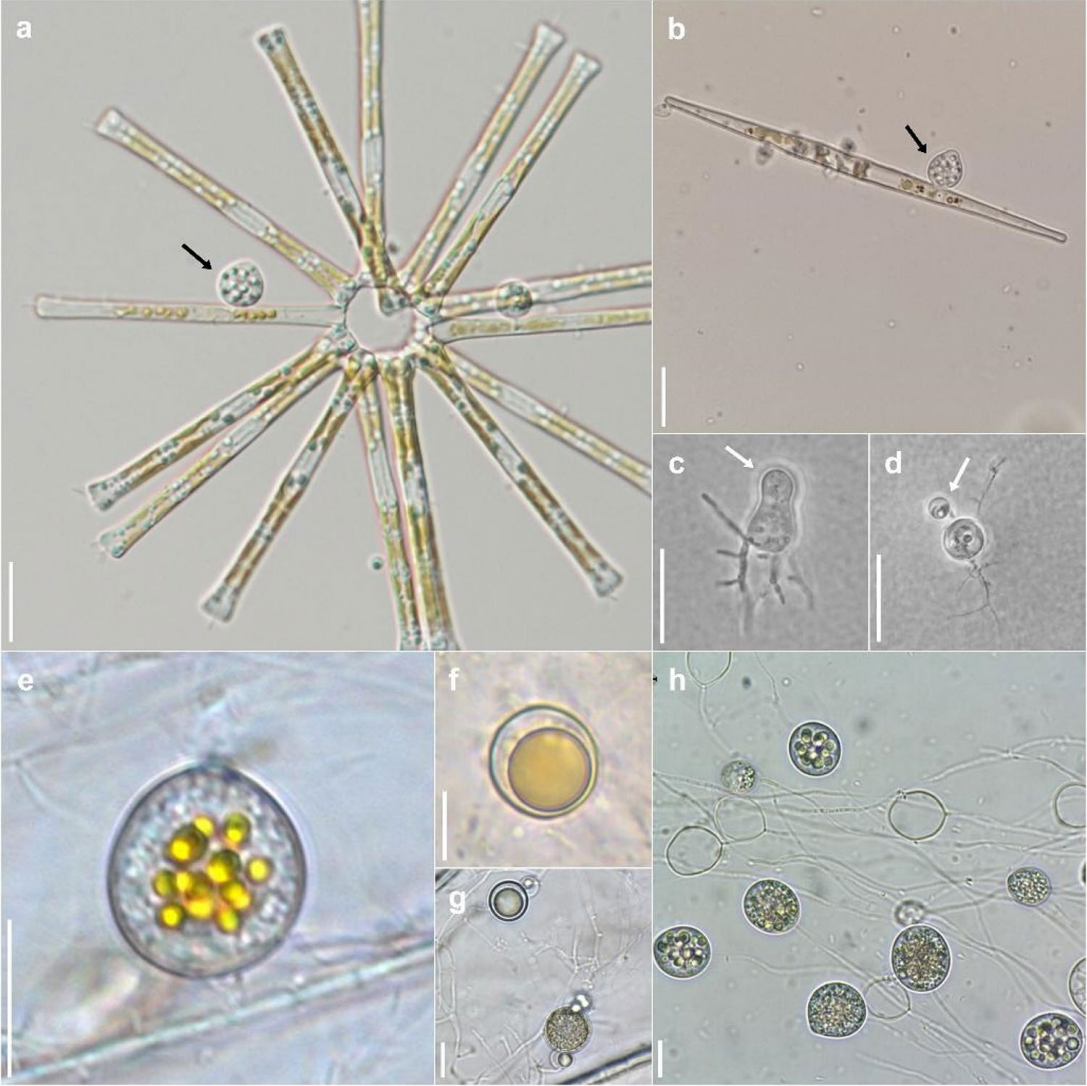
Morphological similarity of *Chytridiomycetes* masks genetic divergence between species and orders. **a** Zoosporangium of *Zygophlyctis asterionellae* (*Zygophlyctidales*) (black arrow) parasitizing the diatom *Asterionella*. **b** Zoosporangium of *Zygophlyctis planktonicum* (*Zygophlyctidales*) (black arrow) parasitizing the diatom *Ulnaria*. **c** Immature zoosporangium and zoospore cyst (white arrow) of *Thoreauomyces humboldtii* JEL095 (*Spizellomycetales*) in PmTG agar medium. **d** Immature zoosporangium and zoospore cyst (white arrow) of *Entophlyctis luteolus* JEL0129 (*Chytridiales*) in PmTG agar medium. **e** Zoosporangium of *Cladochytrium replicatum* CCIBt4014 (*Cladochytriales*) on onion skin. **f** Resting spores of *Cladochytrium replicatum* CCIBt4014 (*Cladochytriales*) on onion skin. **g** Resting spores and intercalary cell of *Cladochytrium tenue* CCIBt4013 (*Chytridiales*) on onion skin. **h** Different zoosporangial stages of *Cladochytrium tenue* CCIBt4013 (*Chytridiales*) in liquid PmTG medium. Scale bars = 10 µm. Photo credits, **a**–**b** Kensuke Seto; **c**–**d** Joyce Longcore, **e**–**h** Gustavo Jerônimo Alves

Although host specificity has tended to be disregarded as a criterion for species identification, it could be important in pathogenic lineages as discussed above. For the well-studied *Zygor*. *planktonicum*, species concepts were revised by a careful re-examination of host range and morphology with molecular techniques (Seto et al. 2020). The host genus is important for species delineation in *Zygophlyctidales*, but it is necessary to say that the degree of host specificity differs depending on lineages of chytrids, ranging from family to species specific (Lepelletier et al. 2014; Seto and Degawa 2018b, a; Ding et al. 2018; Van den Wyngaert et al. 2018a, b; Seto et al. 2020). Most descriptions of parasitic chytrid species are based solely on light microscopic observation. It is necessary to re-examine these species concepts by both cross-inoculation experiments and molecular phylogenetic analyses. Ideally, dual culture-based studies are the best method for examination of species concepts in parasitic chytrids, but it is still a difficult task to establish such cultures. Some culture-independent techniques such as single-cell PCR methods (Kagami et al. in press; Ishida et al. 2015) could be helpful to examine both the host specificity and phylogeny of parasitic chytrids (Fig. 3).

### Species concepts in *Aphelidiomycota*

*Aphelidiomycota* was recently raised to phylum by Karpov et al. (2014b) (as Aphelida) and described as a fungal phylum by Tedersoo et al. (2018). Recent studies either accept aphelids as Fungi or consider them separately in *Opisthosporidia* Karpov, Aleoshin Et Mikhailov (Karpov et al. 2014a, b; James et al. 2020). Within aphelids, four genera have been described (Wijayawardene et al. 2018, 2020): *Aphelidium*, *Amoeboaphelidium*, *Paraphelidium*, and *Pseudaphelidium* containing a total of 15 described species (Wijayawardene et al. 2018, 2020; Letcher and Powell 2019). The morphology, life cycle, ecology, and taxonomy of aphelids have been explored in several studies (Karpov et al. 2014a, b, 2016; Naranjo-Ortiz and Gabaldón 2019; Letcher and Powell 2019; Hurdeal et al. 2021). All described representatives of aphelids are phagotrophic parasites of algae. The life cycle and morphology of aphelids resemble those of rozellids, however the two can be differentiated based on the host (Karpov et al. 2014a, b; Torruella et al. 2018; Letcher and Powell 2019). Aphelids include a characteristic food vacuole with an excretory body, lamellar or tubular cristae, and ciliated or amoeboid cells.

#### Morphology

Species delineation of aphelids using exclusively light microscopy is insufficient, as microscopic features do not provide adequate resolution. For example, *Paraphelidium* is genetically distinct, but morphologically indistinguishable from other aphelid genera (Karpov et al. 2017). All known aphelids share similar life cycle stages, which include zoospores, cysts, and multinuclear plasmodia. Zoospore morphology is important in distinguishing species among genera. Specifically, the zoospores of *Amoeboaphelidium* are amoeboid with highly reduced flagellae. The zoospores of *Paraphelidium* and *Aphelidium* are also amoeboid forming filopodia and lamellipodia. However, the zoospores of *Paraphelidium* can produce subfilopodia from lamellipodia compared to members of *Aphelidium* who do not produce these structures. The zoospores of marine *Pseudaphelidium*, encyst immediately upon release and subsequently release motile zoospores from these cysts. Informative taxonomic characters to distinguish genera include spore size and shape, length of the flagellum, nature of pseudopodia, morphology of sporocyst, size of the residual body in the plasmodium, and presence or absence of a resting spore (Letcher and Powell 2019).

Differences in the morphology within species of the same genus have also been documented (Letcher and Powell 2019). These are taxonomically informative and can be used in species delimitation. Flagellum length and size of cyst, spore, and resting spore are used to characterize species within aphelid genera. For instance, the spore size of *Aphelidium* ranges from 1.5 to 4 μm (Letcher and Powell 2019; Karpov et al. 2020), with the exception of *Aphelidium melosirae*, which produces larger spores (4 × 6 µm). Regarding flagellum length, in *A. tribonematis* it ranges from 6 to 8 µm, while in other members, the flagellum is longer ranging from 9 to 14 µm. For *Paraphelidium*, until now only two species have been discovered and documented (Karpov et al. 2017; Letcher and Powell 2019). Spore size of *Paraphelidium* is around 2–2.5 µm, but differences in spore shape length of flagellum and number of lamellipodia and subfilopodia have also been noted. The flagellum of *Paraphelidium tribonematis* is around 7 µm long, while that of *Paraphelidium letcheri* ranges from 8 to 10 µm. Given that only a few species are known in this genus and in aphelids in general, it is difficult to advocate which morphological characters are taxonomically informative. As more species are discovered and described, it is likely that additional informative characters will be discovered.

#### Molecular phylogeny

The use of molecular phylogenetics has increased significantly in recent fungal taxonomic studies. Molecular phylogeny has helped improve and facilitate taxonomic classification of numerous taxa. Many recent taxonomic studies have incorporated a polyphasic approach using molecular phylogeny, as well as, other criteria (Karpov et al. 2016; Tcvetkova et al. 2019; Seto et al. 2020). For aphelids, molecular phylogenetic analysis is based on SSU rRNA sequences. Using taxa with available molecular sequence data, phylogenetic hypotheses to discriminate species have been proposed. All recently described aphelids have been clearly discriminated using molecular phylogenetics. Genetic distance of DNA sequences can also be informative; however, this should be assessed on a case-by-case basis. For instance, the divergence rate in the SSU rRNA between species in *Aphelidium* is around 15%, *Amoeboaphelidium* 25%, and among the genera *Aphelidium*, *Amoeboaphelidium* and *Paraphelidium* is around 20–25% (Letcher et al. 2017).

### Species concepts in *Rozellomycota*

*Rozellomycota* species are intracellular parasites that grow as naked protoplasts within their hosts. Designation of this phylum was accepted by Tedersoo et al (2018). The *Rozellomycota* consists of *Rozella* species that are mainly endoparasites of water moulds, and *Paramicrosporidium* and *Nucleophaga* that are endonuclear parasites of amoebae. *Rozellomycota* groups in some phylogenies together with the *Aphelidiomycota*, endoparasites of algae, and the *Microsporidia*, mainly endoparasites of animals, at the base of the fungal tree of life (Corsaro et al. 2020), or in other analyses only with *Microsporidia*, while *Aphelidiomycota* diverges later (Torruella et al. 2018; Galindo et al. 2021). Nonetheless, their inclusion within the fungal kingdom is debated. Karpov et al. (2014a, b) included *Rozellomycota* within the *Opisthosporidia* together with aphelids. Environmental surveys have implied a rich biodiversity, which remains poorly characterised (Jones et al. 2011a, b; James et al. 2020). All known species are parasites of algae, water molds (e.g. *Rozella*), crustaceans (e.g. *Mitospodidium*), and amoebae (e.g. *Nucleophaga*, *Paramicrosporidium*) (Corsaro et al. 2020). Identification of *Rozellomycota* and species delimitation often relies on a combination of morphology and molecular phylogeny using the SSU rDNA gene region. *Microsporidia*, a well-known group of intracellular parasites has also been placed within *Rozellomycota*, though this is debated (Tedersoo et al. 2018; Wijayawardene et al. 2018, 2020; Adl et al. 2012; James et al. 2020).

#### Morphology

Morphological characters are informative and crucial for identification of *Rozellomycota*. Use of ultrastructural characters, such as the zoospores, polar filaments, and sporangium among others is common practice. True *Microsporidia* are distinguished by lacking a mitochondrion and by having a spore with a well-developed polar filament (Corsaro et al. 2014; Quandt et al. 2017). Species of *Microsporidia* are difficult to distinguish based on morphological features and host information needs to be considered (Keeling 2009). There are currently over 1300 described species in this group. Herein, *Amphiacantha*, an aquatic genus that parasitizes gregarine protists, is used to illustrate how morphology has been used to describe microsporidian species. Three species are accepted within *Amphiacantha* (Wijayawardene et al. 2018, 2020). Two of these (*Amphiacantha ovalis* and *Amphiacantha attenuate*) were described and introduced by Stubblefield (1955) based on morphological observations. Stubblefield used the characters of the cysts to classify *Amphiacantha* in *Metchnikovellidae* (now the genus is classified in *Amphiacanthidae*). Both species were described as having closely similar gametocysts as *A. longa* and were isolated from the same or closely related species of gregarines. Small differences in the dimensions of the cysts of *A. ovalis* and *A. attenuate* were also noted. *Amphiacantha ovalis* had a higher size average than *A. attenuate*. Another significant observation was the number of gametocysts in the cyst. *Amphiacantha attenuate* had 32–60, while *A. ovalis* had 14–50 and that of *A. longa* was 100. The latter also had significant bigger cysts (70–80 × 4.5 µm). Thus, cyst size can be significant in species delimitation of *Amphiacantha*. However, since only very few species of *Amphiacantha* are known the value of this character may change as more taxa are introduced later on.

*Rozella* species have uniflagellated zoospores, which germinate intracellularly to form a wall-less thallus. Thallus morphology and ultrastructure are used to distinguish *Rozella* species. This genus is differentiated from others by holocarpic, endobiotic, unwalled and inoperculate thallus, one or more discharge papillae (zoospores) with a single posterior flagellum and resting spores, which are thick-walled, smooth and spiny (Letcher et al. 2017; Sparrow 1960). Intrageneric species differentiation takes into account the size of the zoospores, parasite sporangial morphology, and presence of host hypertrophy. For example, the hosts of *Rozella rhizoclosmatii* comprise enlarged host sporangia, while there is absence of hypertrophy in some species including *Rozella apodyae brachynematis*. The morphology of the resting spores also has some value as a taxonomically informative character (Letcher et al. 2017). However, identification based exclusively on morphology can be difficult and at times uninformative. This has been shown with the use of modern molecular phylogeny based on DNA-based sequences.

#### Molecular phylogeny

The SSU rRNA gene is used in molecular phylogenetic analysis of *Rozellomycota* (Corsaro et al. 2016, 2020). This includes the introduction of new species. In most taxonomic research of zoosporic basal fungi, sequence data of related environmental samples are included. This is done in order to account for the low number of taxa currently described, to show the diversity of these fungi and to see if they have already been picked up in environmental surveys. Similarly, to the aphelids, the lack of available data highlights the need for more taxonomic work on these organisms. So far, the large number of sequence data available from environmental samples depicts a great diversity yet to be explored (Corsaro et al. 2016).

### Species concepts in *Neocallimastigomycota*

Although the zoospores of anaerobic fungi were observed in the earliest studies of the rumen ecosystem (Gruby and Delafond 1843) and several were named (as flagellate protozoa) more than a century ago (Braune 1913; Liebetanz 1910), it was only in the 1970s that these cells were recognised as the fungal propagules of fungi, linked to rhizomycelial systems, which grow on ingested feed particles (Orpin 1975, 1976). The anaerobic fungi are classified in the phylum *Neocallimastigomycota* M. J. Powell (Hibbett et al. 2007), class *Neocallimastigomycetes* M. J. Powell (Hibbett et al. 2007). This class contains a single order (*Neocallimastigales*) and a single family (*Neocallimastigaceae*). Nineteen genera are currently recognised within *Neocallimastigaceae* (Table 2), with an additional new genus (*Paucimyces*) has been currently published (Hanafy et al. 2021). All but six of these genera have been named since 2015, the result of an upsurge in research activity relating to these fungi over the past decade, primarily driven by the biotechnological potential of these fungi. These activities have led to the publication of ca. 10 genome sequences and 30 transcriptomes (Solomon et al. 2016; Murphy et al. 2019; Wang et al. 2019).

**Table 2:** List of all named genera and species in class *Neocallimastigomycetes* based on data from Index Fungorum

Genus	Thallus	Flagella	No. spp	Reference	Typus
*Aestipascuomyces*	monocentric	> 16	1	Stabel et al. (2020)	*dupliciliberatus*
*Agriosomyces*	monocentric	< 4	1	Hanafy et al. (2020a, b)	*longus*
*Aklioshbomyces*	monocentric	< 4	1	Hanafy et al. (2020a, b)	*papillarum*
*Anaeromyces*	polycentric	< 4	4(5)	Breton et al. (1990)	*mucronatus*^a^, *contortus*^a^, *elegans*, *[polycephalus]*, *robustus*^a^
*Buwchfawromyces*	monocentric	< 4	1	Callaghan et al. (2015)	*eastonii*
*Caecomyces*	bulbous	< 4	3(4)	Gold et al. (1988)	*[equi],* churrovis^a^, communis, sympodialis
*Capellomyces*	monocentric	< 4	2	Hanafy et al. (2020a, b)	foraminis, elongates
*Cyllamyces*	bulbous	< 4	1(2)	Ozkose et al. (2001)	aberensis, *[icaris]*
*Feramyces*	monocentric	> 16	1	Hanafy et al. (2018)	austinii^a^
*Ghazallomyces*	monocentric	> 16	1	Hanafy et al. (2020a, b)	*constrictus*
*Joblinomyces*	monocentric	< 4	1	Hanafy et al. (2020a, b)	*apicalis*
*Khoyollomyces*	monocentric	< 4	1	Hanafy et al. (2020a, b)	*ramosus*
*Liebetanzomyces*	monocentric	< 4	1	Joshi et al. (2018)	*polymorphus*
*Neocallimastix*	monocentric	> 16	3(6)	Hanafy et al. (2020a, b)	*frontail*^a^*, cameroonii, californiae*^a^*, [hurleyensis], [patriciarum], [variabilis]*
*Oontomyces*	monocentric	< 4	1	Dagar et al. (2015)	*anksri*
*Orpinomyces*	polycentric	> 16	2(3)	Barr et al. (1989)	*bovis, intercalaris, [joyonii]* ^a^
*[Paucimyces]*	polycentric	< 4	1	[Hanafy et al. (2020a, b)]	*[polynucleatus]*
*Pecoramyces*	monocentric	< 4	1	Hanafy et al. (2020a, b)	*ruminantium*^a^
*Piromyces*	monocentric	< 4	4(10)	Gold et al. (1988)	*communis, [citroii], cryptodigmaticus, [dumbonicus],*
					*finnis*^a^*, [irregularis], [mae], [minutus], rhizinflatus*^a^*, [spiralis]*
*Tahromyces*	monocentric	< 4	1	Hanafy et al. (2020a, b)	*munnarensis*
			32(45)		

^a^Indicates species where genome and/or transcriptome data are available (not listed are eight genomes/transcriptomes of unnamed *Piromyces* spp.)

**Fig. 4:**
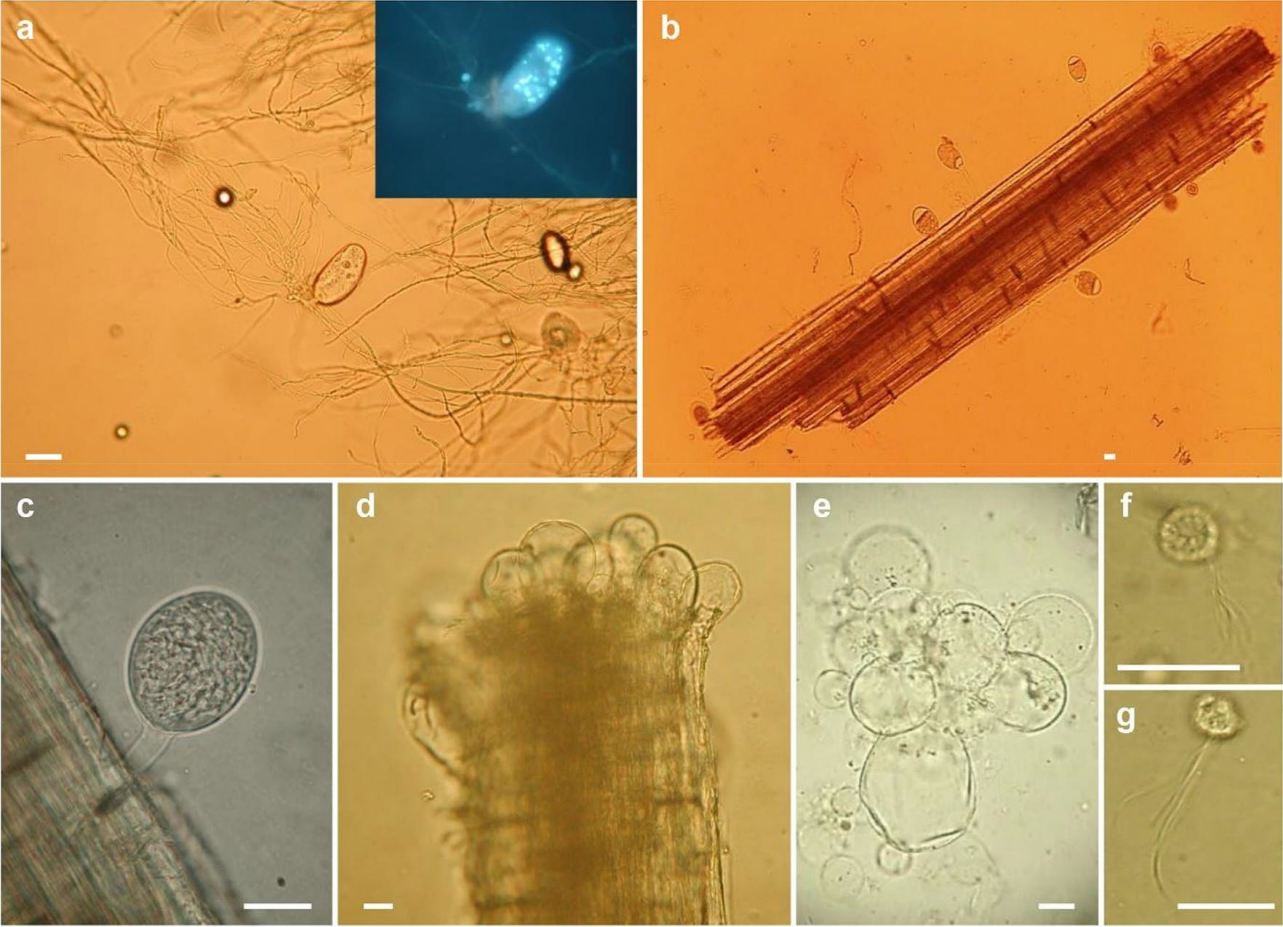
Anaerobic fungi (*Neocallimastigomycota*). **a** *Buwchfawromyces eastonii* exhibiting monocentric growth morphology (inset image of the sporangium stained with DAPI to show the nuclei of the developing zoospores within the zoosporangium). **b**, **c** *Piromyces* sp. sporangia emerging from a fragment of wheat straw. **d** Thalli of *Caecomyces* sp. growing from the end of a forage particle. **e** Detailed view of a thallus of *Caecomyces* sp. showing the multiple bulbous holdfasts. **f**, **g** Multiflagellate zoospores of *Neocallimastix frontalis*. Scale bars = 20 µm. Images by Gareth Griffith and Tony Callaghan

Stable and distinctive morphological characters permitting differentiation of the anaerobic fungi are meagre, specifically: the formation of a bulbous holdfast (*Caecomyces* and *Cyllamyces*) or a rhizoidal thallus (all other genera); the formation of a polycentric thallus bearing several sporangia with nuclei present in the rhizoids (*Anaeromyces*, *Orpinomyces*, *Paucimyces*) or more limited monocentric anucleate thallus bearing a single sporangium (all other genera); multiflagellate zoospores (bearing > 16 flagella; *Aestipascuomyces*, *Feramyces*, *Ghazallomyces*, *Neocallimastix*, *Orpinomyces*) or uniflagellate zoospores (rarely 2–4 flagella; all other genera). It is noteworthy that the formation of multiflagellate zoospores in these genera appears to be a unique trait not found elsewhere amongst opisthokonts, with phylogenetic reconstruction suggesting that this trait arose once within the anaerobic fungi, with a single reversion to uniflagellate zoospores (*Pecoramyces*) (Hanafy et al. 2017, 2018, 2020b; Stabel et al. 2020). Morphological features of anaerobic fungi (*Neocallimastigomycota*) are illustrated in Fig. 4.

The majority (11) of the currently recognised genera form monocentric thalli and uniflagellate zoospores. The first of these was isolated and characterised by Orpin (1977) and later named as *Piromyces* by Gold et al. (1988). However, it is likely that some of the nine species later named within *Piromyces* prior to the advent of DNA barcoding should more correctly have been placed in one of the other 10 morphologically indistinguishable genera. However, the absence of physical type material or ex-type cultures for six of these species prevents exploration of this possibility.

In contrast to other eukaryotes inhabiting the mammalian digestive tracts, notably the ciliate protozoa (Cedrola et al. 2017; Newbold et al. 2015), the anaerobic fungi show remarkably little host specificity. Possible exceptions include *Oontomces anksri* (pseudoruminant camellids) (Dagar et al. 2015), *Piromyces finnis* (horse) (Hanafy et al. 2020b) and *Ghazallomyces constrictus* (axis deer) (Hanafy et al. 2020a). With the exception of Liggenstoffer et al. (2010) and Hanafy et al. (2020a), who used DNA metabarcoding to assess rumen fungal populations in diverse wild, captive and domesticated animals, the distribution of anaerobic fungi has mostly focused on domesticated hosts. The lack of host specificity is likely due to the efficiency with which anaerobic fungi disperse between hosts (Becker 1929). These are known to survive for considerable periods under aerobic conditions (McGranaghan et al. 1999) but the aerotolerant propagules, which are occasionally observed have hitherto defied detailed investigation (Brookman et al. 2000).

DNA-based identification of anaerobic fungi initially focused on the ITS1 spacer region but the high level of intragenomic variation, sometimes > 10% sequence divergence between the ITS1 repeats of a single isolate (Callaghan et al. 2015), necessitated the cloning of amplicons prior to sequencing, as well as problematic sequence alignment. However, sequencing of the D1/D2 subunits of the LSU locus has permitted robust phylogenetic reconstruction and provides clear species delimitation (Dagar et al. 2011). Most recently, the use of PacBio SMRT technology has permitted DNA metabarcoding of gut fungal communities from environmental samples using a combined ITS1/2 and D1/D2 LSU amplicon (Hanafy et al. 2020a). Not only does this permit reliable linkage of sequences to species names via the LSU region but also the identification of published sequences from unidentified environmental sequences, which comprise mostly ITS1 only. Phylogenetic reconstruction based on ITS1 and informed by predicted secondary structure had previously identified a number of hitherto unidentified clades of anaerobic fungi (Koetschan et al. 2014). Several of these have since been named from pure cultures and based on the PacBio sequencing approach developed by Hanafy et al. (2020a), at least 10 additional genera remain unnamed beyond the 20 hitherto formally named. There is no reason to believe that these additional taxa are refractory to axenic cultivation.

**Fig. 5:**
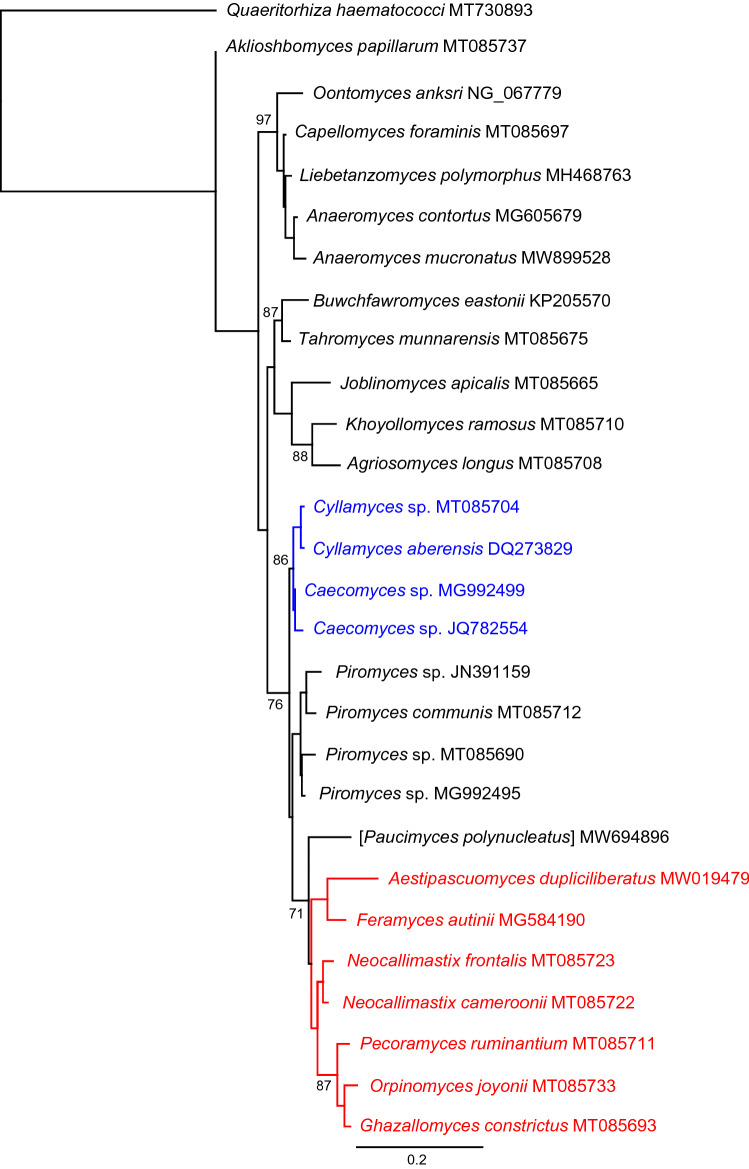
Phylogenetic reconstruction using Maximum Likelihood based on partial sequences of 28S rRNA locus showing evolutionary relationships within *Neocallimastigomycota*. Blue indicates bulbous clades and red indicates clade with multiflagellate zoospores (except *Feramyces*). Scale bar indicates substitutions per site and salient bootstrap support values (1000 replicates) are shown at nodes. The outgroup (*Quaeritorhiza*) is a chytrid parasite of *Haematococcus pluvialis* (Chlorophyta)

There are currently 45 named species but only 32 of these are clearly correct at present. Some additional names exist in the literature, most puzzlingly *Piromyces equi* for which no formal description exists beyond its name but which has been studied in numerous physiological publications [e.g. (Nagy et al. 2007; Poidevin et al. 2009)]. A phylogeny representing evolutionary relationships within *Neocallimastigomycota* is shown in Fig. 5.

### Ecology and distribution of *Mucoromycota*

#### Ecology

The first taxonomic studies of the phylum *Mucoromycota*, whose species were treated within the traditional phylum *Zygomycota*, were in the late nineteenth century, mainly in France (van Tieghem and Le Monnier 1873; van Tieghem 1875, 1878; Bainier 1882), Germany (Fischer 1892; Schröter 1889) and the USA (Sumstine 1910). In the early twentieth century, subsequent studies were conducted in Switzerland (Lendner 1908), USA (Blakeslee 1905; Thaxter 1922) and Norway (Hagem 1907, 1910), followed by studies in Germany (Zycha 1935; Zycha et al. 1969) and Pakistan (Mirza et al. 1979). However, since the 1960s, there has been a considerable increase in information regarding the morphological taxonomy of the phylum *Mucoromycota*, which is mainly attributed to taxonomic revisions by Hesseltine and Fennell (1955) (USA, genus *Circinella*), Hesseltine and Ellis (1961, 1964, 1966) (USA, *Absidia*), Schipper (1973, 1975, 1976, 1978a, 1978b, 1984) (The Netherlands, *Mucor*, *Rhizomucor*, and *Rhizopus*), Benny and Benjamin (1975, 1976), Benny et al. (1985) (USA, *Thamnidiaceae*), Benjamin (1979) (USA, *Endogonaceae*), Zheng and Chen (2001) (China, *Cunninghamella*), Meyer and Gams (2003) (Australia/The Netherlands, *Umbelopsis*), Zheng et al. (2007) (China, *Rhizopus*), Walther et al. (2013) (Germany, *Mucorales*), Alastruey-Izquierdo et al. (2010) (Spain, *Lichtheimia*), and Wagner et al. (2019) (Germany, *Mucor circinelloides* complex).

In most previous studies, the classification of *Zygomycota* has been performed according to morphological characteristics; however, the advent of molecular biology, which was a major milestone in science and the taxonomic characterization of this phylum, has permitted the regrouping of zygomycotan taxa into new phyla, classes, orders, and families (Voigt 2012a, b; Hoffmann et al. 2013; Spatafora et al. 2016; Tedersoo et al. 2018). Among the seven phyla of zygosporic fungi, *Mucoromycota* is by far the most studied and comprises the largest number of species (354 spp.) that are distributed among the orders *Mucorales* (303 spp.), *Endogonales* (34 spp.), and *Umbelopsidales* (17 spp.) (Wijayawardene et al. 2020; www.speciesfungorum.org).

Benny and Benjamin (1991) recognized the order *Mucorales* and the families *Choanephoraceae*, *Cunninghamellaceae*, *Gilbertellaceae*, *Mucoraceae*, *Mycotyphaceae*, *Phycomycetacea*e, *Radiomycetacea*e and *Thamnidiaceae* based on their morphological characteristics. At that time, *Endogonales* only covered the family *Endogonaceae*, and *Umbelopsidales* (Spatafora et al. 2016) was not yet proposed; moreover, their species were allocated in the *Mortierellale*s (Cavalier-Smith 1998). However, molecular analyses conducted by O’Donnell et al. (2001), Voigt and Wöstemeyer (2001), Meyer and Gams (2003), Voigt (2012a, b), and Hoffmann et al. (2013) provided new insights into the classification of Mucoromycota along with the establishment of new families, while others were disregarded. Presently, the following 3 orders and 17 families are accepted in the phylum *Mucoromycota*: (1) *Mucorales*: *Backusellaceae, Choanephoraceae*, *Cunninghamellaceae*, *Lentamycetaceae*, *Lichtheimiaceae*, *Mucoraceae*, *Mycocladaceae*, *Mycotyphaceae*, *Phycomycetaceae*, *Pilobolaceae*, *Radiomycetaceae*, *Rhizopodaceae*, *Saksenaeaceae*, and *Syncephalastraceae*; (2) *Endogonales*: *Densosporaceae*, and *Endogonaceae*; and (3) *Umbelopsidales*: *Umbelopsidaceae* (Wijayawardene et al. 2020; Hoffmann et al. 2013; Desirò et al. 2017). Most species belonging to the *Mucoromycota* are saprobic and found in soil (Benny et al. 2016), although some taxa of *Mucorales* and *Umbelopsidales* are endophytes (Bezerra et al. 2018; Sarsaiya et al. 2019; Rashmi et al. 2019). *Endogonales*, in addition to being saprobic, are ectomycorrhizal symbionts of plants as well as endophytes (Desirò et al. 2017; Bonafante and Venice 2020). Species of *Mucorales* can also be commonly isolated from the excrement of animals, such as herbivores and rodents (Benny 2008, 2012; Santiago et al. 2011; Melo et al. 2020), dead vegetables, and stored grains (Hoffmann et al. 2013). Moreover, the presence of these fungi in different substrates reflects their ecological importance in biodegradation processes, primarily in decomposition and nutrient cycling (Dix and Webster 1995; Bills et al. 2004; Richardson 2009). Facultative parasitic species of plants (Benny et al. 2014) and animals, including humans, are known to cause mucormycosis (Kamei 2000; Jacobsen 2019). Some genera are facultative mycoparasites, such as *Chaetocladium*, *Dicranophora*, *Spinellus*, and *Syzygites* (Zycha et al. 1969; Voglmayr and Krisai-Greilhuber 1996; Kovacs and Sundberg 1999; Benny 2005), whereas *Sporodiniella* is a facultative insect parasite (Evans and Samson 1977).

In *Mucoromycota, Pilobolus* is the unique obligatory coprophilous genus and, therefore, exhibits morphological characteristics that allow its growth on excrement as well as its dispersion from this substrate. These include positive phototropism mechanisms with active expulsion of the adhesive sporangia, sporangiospores that survive the digestion of animals and the capacity to grow under relatively high pH conditions (Dix and Webster 1995). As obligate coprophiles, *Pilobolus* species are difficult to grow on artificial media, even on dung agar or culture media supplemented with hemin. We have been trying to cultivate different species of *Pilobolus* over the past few years and have observed low growth and smaller morphological structures compared to those that grow directly on dung. In addition, most of the strains that can be grown on artificial media do not survive through sequential transfers to other plates (Foos et al. 2011). Other genera, such as *Pilaira*, *Benjaminiella*, *Chaetocladium*, *Cokeromyces*, *Ellisomyces*, *Phascolomyces*, *Phycomyces*, *Thamnostylum*, *Utharomyces*, and *Zychaea*, are mostly coprophilous, but some may include one or more non-coprophilic species or records (Krug et al. 2004).

**Fig. 6:**
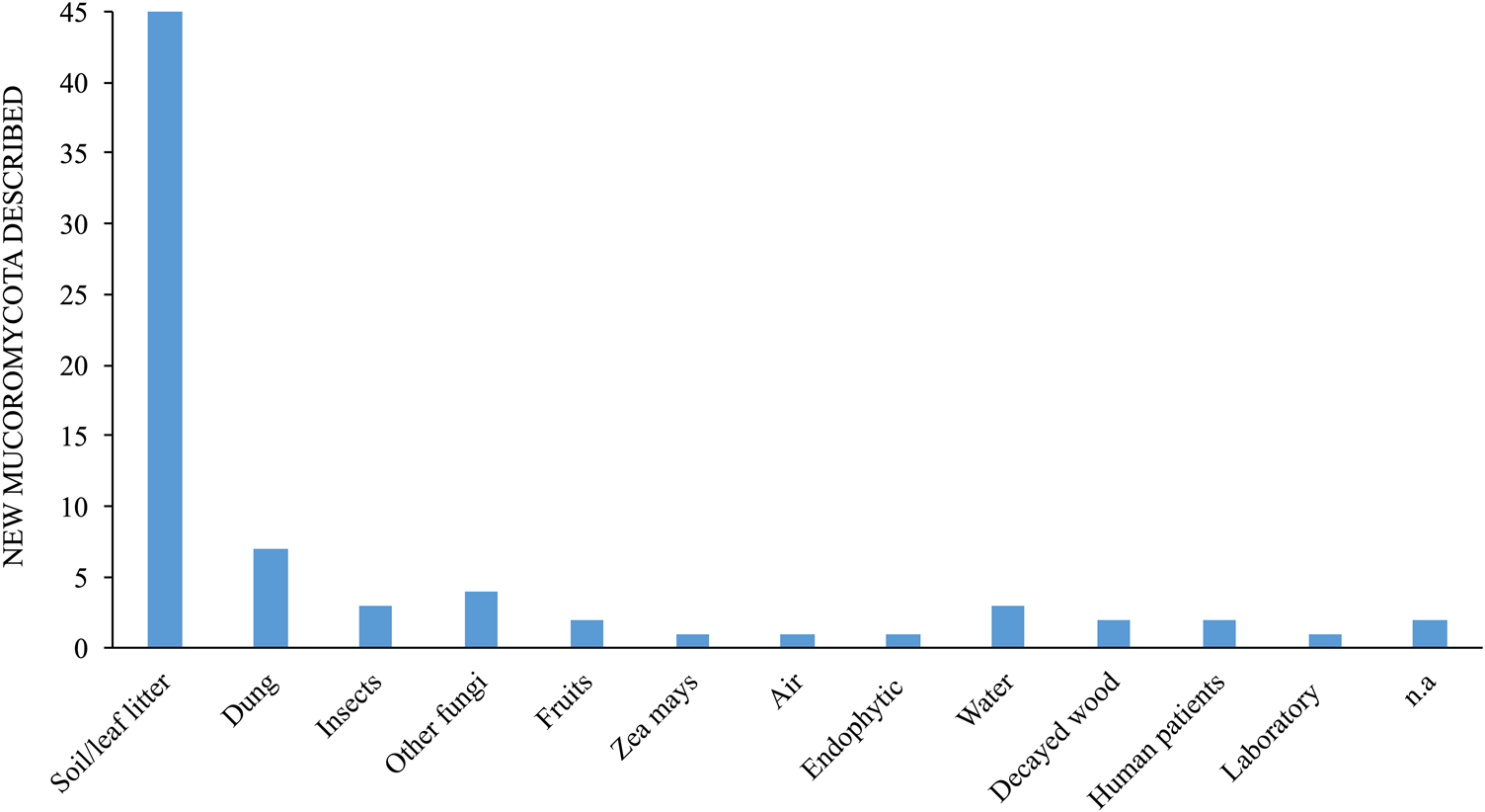
Number of *Mucoromycota* species described for the first time from 2015 to 2020 (until October 16) in different substrates

Unfortunately, data on the ecology of *Mucoromycota* are scarce. Considering the abundance of fungi in the soil environment at some stage in their life-cycle (Bridge and Spooner 2001), it is not surprising that this substrate is preferred by researchers for the isolation of *Mucoromycota*. For example, according to the Index Fungorum database (http://www.indexfungorum.org), of the 74 newly identified species of *Mucoromycota* described for the first time from 2015 to 2020 (until October 16), 45 (60.8%) were isolated from either soil or leaf/litter, followed by some from animal excrements, along with the reports from other fungi, insects, and water. Only a few species were isolated from decayed wood, human patients, fruits, air, *Zea mays*, and as laboratory contaminants (Fig. 6). Thus, the information regarding the taxonomy and ecology of *Mucoromycota* could advance considerably if there were more taxonomists willing to do inventories of these fungal species, specifically in poorly or unexplored habitats/hosts (Hawksworth and Rossman 1997; Hawksworth 2001a, b; Aime and Brearley 2012), thereby revealing unforeseen ecological interactions. For example, the well-known saprotrophic fruit parasite, *Gilbertella persicaria* (Mehrotra 1963a, 1963b; Guo et al. 2012; Pinho et al. 2014), was found infecting the black tiger shrimp (*Penaeus monodon*) (Karthikeyan and Gopalakrishnan 2014), while *Actinomucor elegans*, a saprotrophic soil fungus, which is occasionally involved in causing mucormycosis, has been reported to infect chafer beetle (Karimi et al. 2015). This fungus has also been found in a mutualistic association with *Abutilon theophrasti* roots (Kia et al. 2014). Interestingly, a high number of specialized operational taxonomic units of *Mucor* has been detected in decomposing *Picea abies* wooden blocks (Gómez-Brandón et al. 2020). In general, since mucoralean species do not use cellulose or lignin but use less complex and soluble sources of carbon and nitrogen (Benny 2012), they are probably using simple sugars produced by other fungi decomposers of cellulose or lignin. Interestingly, Gómez-Brandón et al. (2020) demonstrated the association of *Mucor* spp. with bacterial endosymbionts of the order *Burkholderiales*. This type of endosymbiotic interaction has previously been reported in *Rhizopus microsporus*, a well-known plant pathogen, which hosts *Mycetohabitans rhizoxinica* and *M*. *endofungorum*. These bacteria supplement the fungus with toxins that are the causative agent of plant disease and are involved in the regulation of asexual and sexual reproduction in the fungus (Partida-Martinez et al. 2007a,b). Other interesting and unusual ecological findings with respect to *Mucoromycota* that require further studies are as follows: pathogenic nature of *Syncephalastrum* sp. in gardens of leaf-cutting ants (Barcoto et al. 2017); *Mucor racemosus* as part of the lichen-associated mycobiota (Tripathi et al. 2014; Lagarde et al. 2018); and frequent and occasional human mucormycosis-causing agents, *Apophysomyces variabilis* (Prakash et al. 2016) and *Actinomucor elegans* (Dorin et al. 2017), respectively, which have been observed to be a part of the green turtle nest mycobiota, where both the fungal species can be considered novel pathogens for the turtles (Candan 2018). *Mucor abundans*, *Umbelopsis angularis*, *U*. *isabelina*, *U*. *ramanniana* and a new putative species of *Apophysomyces* have been found to be associated with the millipede fungivore *Brachycybe lecontii* (Macias et al. 2019). Additionally, zygospores of the two new mucoralean endoparasites, *Mucor lilianae* and *M*. *rudolphii*, have been found to be present in the basidiomas of *Hysterangium* spp. (Voglmayr and Clémençon 2015).

#### Distribution

Although several studies on the taxonomy and classification of *Mucoromycota* have been conducted (including the description of new genera and species), the species inventories of this phylum in different domains and substrates existing worldwide are not only limited but also temporally and spatially distributed. Some of these studies have been conducted in Brazil (Schoenlein-Crusius et al. 2006; de Souza et al. 2008, 2017; Santiago et al. 2011, 2013; Lima et al. 2015, 2016, 2018; Melo et al. 2020), Chile (Oscar Martínez and Eduardo Valenzuela 2003), China (Chen et al. 2007), France (Mousavi et al. 2018), Indonesia [Boedijn 1958], Iran (Ziaee et al. 2016), Malaysia (Loh et al. 2001; Lee et al. 2018), Mexico (Cruz-Lachica et al. 2018), Pakistan (Mirza et al. 1979), the UK (Campbell 1938), and the USA (Christenberry 1940). Therefore, whether the majority of the known species belonging to the phylum *Mucoromycota* are cosmopolitan cannot be confirmed. The genera commonly isolated from soil and decomposing vegetables, those causing food spoilage (e.g. *Absidia*, *Cunninghamella*, *Rhizopus*, *Gongronella*, *Syncephalastrum*, and *Umbelopsis*), plant pathogens (e.g. *Rhizopus*, *Gilbertella*, and *Choanephora*), and the causal agents of mucormycosis (e.g. *Apophysomyces*, *Rhizopus*, *Lichtheimia*, *Mucor*, *Rhizomucor*, and *Saksenaea*) are more frequently mentioned, and in most cases, they can be considered cosmopolitan. However, even within these genera, the distribution of some species remains poorly documented. For example, *Cunninghamella septata* has only been isolated in China (Zheng and Chen. 2001), and *C*. *clavata* (Zheng and Chen. 1998) has only been reported to occur in China and Brazil (Alves et al. 2017). *Absidia idahoensis*, and *A*. *macrospora* have been reported once in the USA (Hesseltine et al. 1990) and Czech Republic (Váňová 1968), respectively, and *Isomucor trufemiae* has only been reported in Brazil (de Souza et al. 2012; de Lima et al. 2020). The monospecific *Halteromyces* and *Chlamydoabsidia* are rare. While the former species has only been reported in Australia (Shipton and Schipper 1975), *Chlamydoabsidia* has been reported both in the USA (Hesseltine and Ellis 1966) and India (Behera and Mukerji 1985). *Hyphomucor* seems to be restricted to India, Malaysia, Sri Lanka (Schipper 1986), Nepal (Mikawa 1988), and Japan, while *Ambomucor* has been recently isolated from China (Zheng and Liu 2013) and the USA (MGnify 2020). *Rhizopodopsis* is also rare and has only been isolated in Indonesia (Boedijn 1958), while *Dicranophora*, *Spinellus*, and *Syzygites* seem to have diverse global distributions. While *Dicranophora* was especially reported from North America and Europe (Benny 2005), *Spinellus* was reported all across the Northern hemisphere (Ueda 2020; van Tieghem 1875; Ling-Young 1930; Indoh 1961; Ellis and Hesseltine 1962). *Syzygites* has been shown to be distributed worldwide (GBIF 2019). The facultative insect parasite, *Sporodiniella*, has only been reported in the warmer regions ranging from Ecuador (Evans and Samson 1977), Taiwan (Chien and Huang 1997), Japan (Orihara 2020) to Papua New Guinea (CABI 2020). *Endogone* is the best-known cosmopolitan species (Cannon and Kirk 2007). *Peridiospora spinosa* seems to be restricted to Brazil (Goto and Maia 2006), and Taiwan (Wu and Lin 1997). *Sclerogone* is restricted to Australia (Warcup 1990). *Jimgerdemannia* has been found in Australia, Japan, Europe and North and Central America (Desirò et al. 2017; Yamamoto et al. 2020). *Vinositunica* is a newly described genus reported in Japan (Yamamoto et al. 2020).

Many genera of *Mucoromycota* grow only or are more common on animal dung, and therefore, data on these coprophilous fungi are still insufficient, which is probably because animal dung may not be “attractive” for many researchers. Additionally, many of these coprophilous fungi do not exhibit any economic potential, and therefore, they may be of minimal interest to researchers. *Pilobolus*, *Pilaira* and *Thamnostylum* have been reported worldwide (Benny and Benjamin 1975; Cannon and Kirk 2007), while *Utharomyces* was reported in Africa, Bahamas, Brazil, Ghana, France, India, Indonesia, Mexico, Panama, Republic of China, the USA, and Venezuela (Alves et al. 2020; MGnify 2017, 2019). *Benjaminiella* (India, Mexico, Spain, and the USA) (Kirk 1989), *Ellisomyces* (the USA) (Hesseltine and Anderson 1956; Benny and Benjamin 1975), *Fennellomyces* (Australia, India, Malaysia, and the USA) (Benny and Benjamin 1975; Mirza et al. 1979; CABI 2020), *Phascolomyces* (China, Indonesia, and Panama) (Boedijn 1958; Benny and Benjamin 1976), and *Zychaea* Benny & RK Benj. (Mexico) (Benny and Benjamin 1975) exhibit restricted distribution. *Chaetocladium*, a parasitic genus of other *Mucorales*, grows better at low temperatures, and therefore, it is more commonly found in temperate countries, such as the Austria, China, Germany, Norway, Portugal, Spain, Sweden, the Netherlands, and United Kingdom (Zycha et al. 1969; Benny and Benjamin 1976). According to Benny (2005), this genus can be found during winters only in the hottest parts of the USA.

Estimates of the number of fungal species on a global scale range between 712,000 to 13.2 million species (Hawksworth 1991, 2001a, b; Schmit and Mueller 2007; Blackwell 2011; Wu et al. 2019). Hawksworth and Lücking (2017) estimated between 2.2 and 3.8 million fungal species, of which only 135,110 have been described up to 2019 (Catalog of Life: http://www.catalogueoflife.org/annual-checklist/2019/). Regrettably, considering the limited number of fungal taxonomists worldwide and the average rate of 2000 species described per year (Hawksworth and Lücking 2017), the task of cataloging all fungal species may take approximately 1430 years at the current rate of progress (Lücking 2020). Undoubtedly, the majority of known fungal species belong to the sister phyla *Ascomycota* and *Basidiomycota*, with only a small percentage of basal fungal species described, including the ones belonging to *Mucoromycota* (www.indexfungorum.org). Therefore, an important question that needs to be addressed is whether the *Mucoromycota* has been more neglected than other phyla of fungi sensu stricto with respect to descriptions of new species. Prior to 2008, nearly 98,000 fungal species were described (Kirk et al. 2008), of which 245 spp. belonged to *Mucoromycota* (a ratio of 400:1). The same ratio was observed eleven years later, based on the Catalogue of Life (2019) that estimated 135,110 known species of fungi along with 338 species belonging to the phylum *Mucoromycota*.

**Fig. 7:**
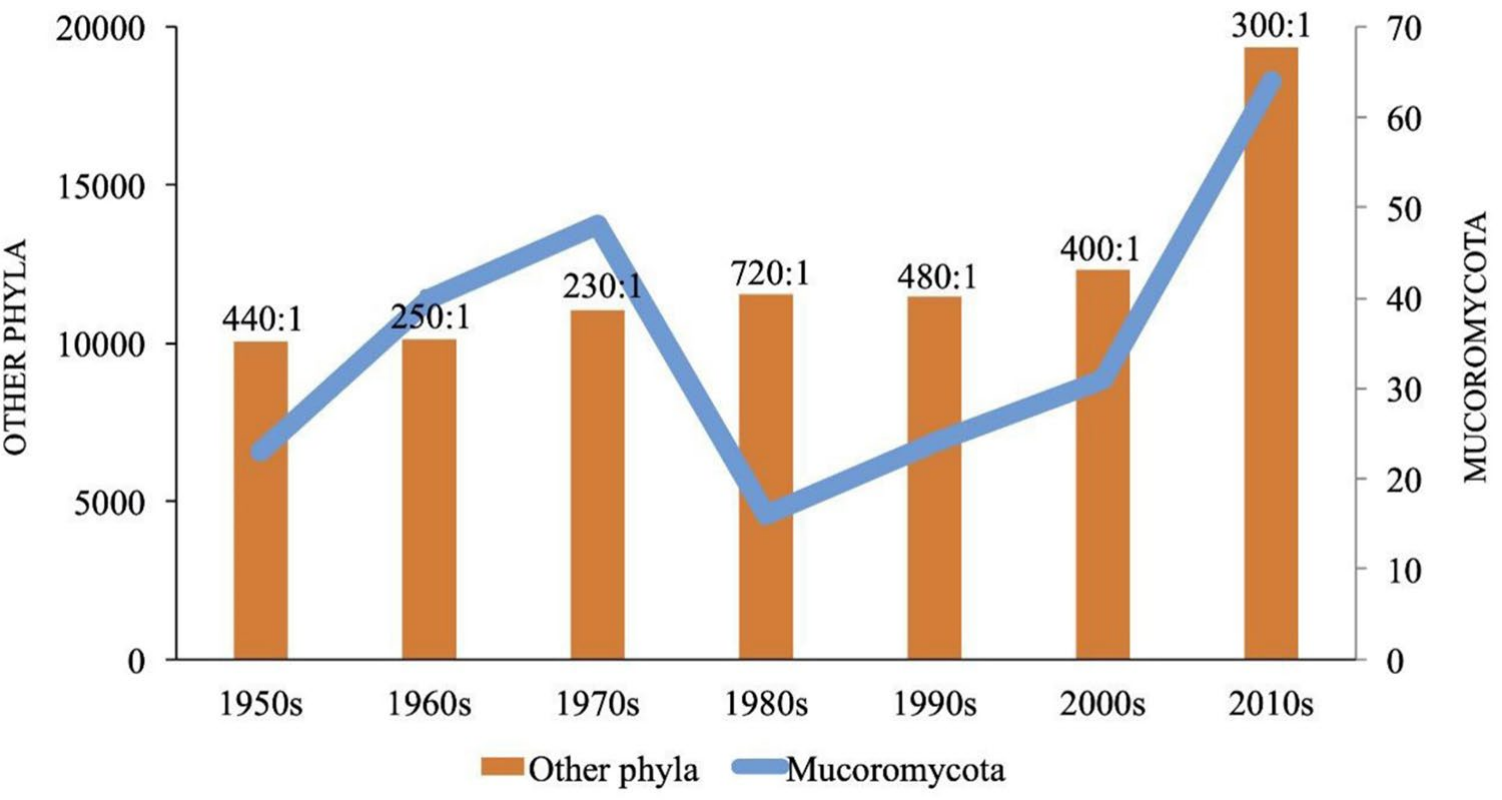
Number of species of *Mucoromycota* and other phyla described for the first time per decade from 1950 to 2019. Only valid species are considered, thereby excluding new combinations. The bars indicate the ratio of other phyla: *Mucoromycota* species described in each decade

**Fig. 8:**
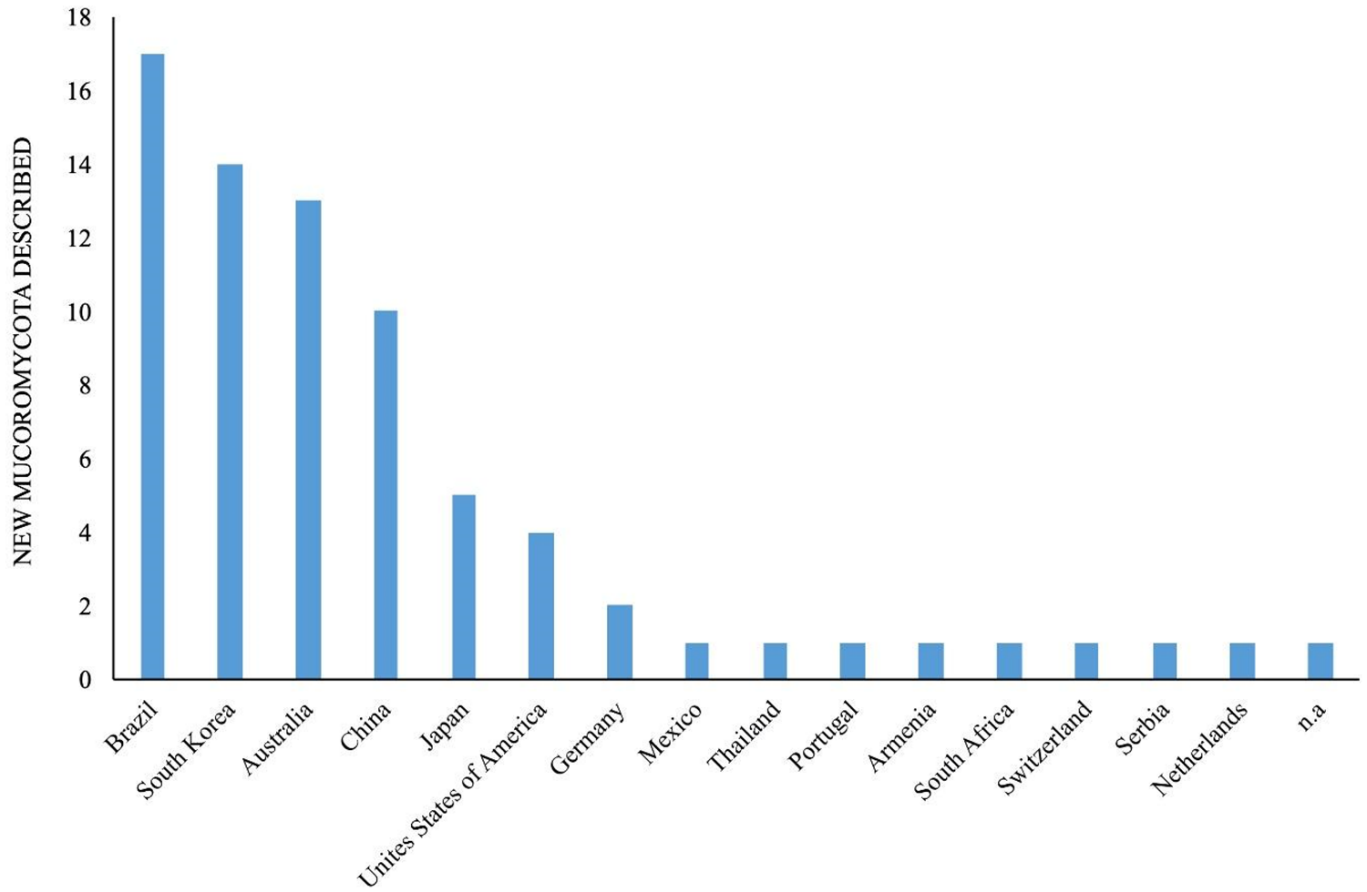
Number of *Mucoromycota* species described for the first time from 2015 to 2020 (until October 16) in different countries

To accurately address the aforementioned question, we expanded our research and retrieved data regarding all new fungal species that were described for the first time between 1950 and 2019 using the Index Fungorum database (http://www.indexfungorum.org). Only valid species were considered, and new combinations were excluded (Fig. 7). In the 1960s and 1970s, 40 and 48 species of *Mucoromycota* were described, with other phyla: *Mucoromycota* ratios of 250:1 and 230:1, respectively. However, the number of new species identified in this phylum markedly reduced in the 1980s (16 spp; ratio of 720:1), followed by a successive increase in the following three decades. The 2010s should be highlighted for the identification of the largest number of mucoromycotan species described since the 1950s (64 spp. until 2019) with other phyla: *Mucoromycota* ratio of 300:1. Moreover, the number of species of other phyla described was also the highest (19,421 spp.). This can probably be attributed to the increased use of molecular biology techniques for species identification (Hawksworth and Lücking 2017; Cheek et al. 2020). Interestingly, considering the average number of species described in the past 70 years, we could estimate on other phyla: *Mucoromycota* ratio of 350:1 (data not shown), which is lower than the ratio projected in 2019 (400:1), based on the Catalog of Life (2019). Therefore, it appears that *Mucoromycota* have not been neglected more than other fungal phyla in terms of description of new species, at least in the past 70 years. So why is the knowledge of distribution of this phylum still so limited? A possible reason is that studies on this phylum have been conducted by restricted groups of taxonomists belonging to just a few countries. Therefore, the number of known species could probably be markedly higher and distribution data could be more accurate than at present if more expert taxonomists become interested in surveying these basal fungi. In addition, description of many new species of *Mucoromycota* as well as information regarding distribution of some taxa are based only on the sources provided by culture collections and clinical studies (Walther et al. 2019). Interestingly, recent data seem to be encouraging since increasing number of taxonomists have dedicated their research to describing new species of *Mucoromycota* over the last few years. For example, between 2015 and 2020 (until October 16), 13,218 species of other fungi and 74 species of *Mucoromycot*a have been described (ratio of 178:1); however, these studies are still restricted to only a few countries. Figure 8 shows that among the 74 newly described species of *Mucoromycota* between 2015 and 2020 (until October 16), 54 (74%) were isolated from Brazil, South Korea, Australia, and China, while the rest were identified from 11 other countries. This confirms that only a few prolific taxonomists are concentrated in some countries, and in many cases, they are concentrated in one region (state or province) of a single country. For example, all of the 17 new species of *Mucoromycota* described in Brazil in the last five years were isolated by the same research group in the state of Pernambuco. The same has been observed in South Korea and Australia. This alarming situation demands attention for the urgent need to train new taxonomists to discover new species of *Mucoromycota* before they become extinct.

In addition to the poorly investigated or unexplored ecosystems, such as tropical areas and diversity hotspots, new species of *Mucoromycota* are also expected to be delimited from species complexes, specifically in the non-revised genera, such as *Absidia*, *Mucor* and *Syncephalastrum*, based on their morphological and molecular features. Wagner et al. (2019), for example, identified five new *Mucor* species after reviewing the *Mucor circinelloides* complex. Moreover, information regarding the distribution of *Mucoromycota* may be significantly enhanced by using data generated from environmental sequence studies. This may also reveal a covert diversity, as observed by Tedersoo et al. (2017), who analysed global soil DNA samples and fungal ITS2 dataset from 365 sites in 38 countries, following which they identified unclassified fungal species phylogenetically related to *Endogonales* and *Umbelopsidales*.

### Impact of *Chytridiomycota*

#### Phytoplankton-infecting chytrids

Next to saprotrophic chytrids, numerous facultative and obligate parasitic forms, which infect diverse plants and microalgae, can be found in a broad range of terrestrial and aquatic environments (Voigt 2012b; Voigt et al. 2013; Frenken et al. 2017). Moreover, chytrids were found to dominate fungal communities in high-elevation soils and link aquatic and terrestrial ecosystems in alpine regions unsuspectedly (Freeman et al. 2009). Spread and propagation take place mainly asexually via free-swimming zoospores, that settle on a host, penetrate the cell and develop rhizoids to extract its nutrients while developing into sporangia, which again release new zoospores upon maturation (e.g. Ibelings et al. 2004).

In studies investigating marine and freshwater plankton communities, chytrids remained almost unrecognized for many decades. This was not only due to the small size of their infectious stages (zoospores), which is in the range of nanoplankton, but also due to their inconspicuous morphological features, which caused several misidentifications as bacterivorous flagellate protozoa (Lefèvre et al. 2007; 2008). In recent years, however, molecular culture-independent surveys have revealed an unexpected widespread occurrence and high abundance of several chytrid lineages (Lefèvre et al. 2012; Wurzbacher et al. 2014; Comeau et al. 2016; Hassett and Gradinger 2016; Hassett et al. 2017). Based on high-throughput sequencing of the hypervariable region V4 of the SSU rRNA gene, chytrids have been documented for example in temperate and polar marine ecosystems, where they made between 38 and 93% of all fungal sequences (Comeau et al. 2016). Comparable results were obtained for freshwater environments using full-length rRNA operon amplicon metabarcoding (Heeger et al. 2018).

Those findings raised the interest of plankton researchers and today many chytrids from diverse orders (e.g. *Chytridiales*, *Gromochytriales*, *Lobulomycetales*, *Mesochytriales*, *Polyphagales*, *Rhizophydiales*, *Synchytriales*) are morphologically identified as lethal parasites across all the major phytoplankton groups (e.g. cyanobacteria, diatoms, dinoflagellates, chlorophytes; Sparrow 1960; Lepelletier et al. 2014; Gutiérrez et al. 2016; Van den Wyngaert et al. 2018a, b). So far, studies quantifying the ecological significance of phytoplankton-infecting chytrids in aquatic environments are scarce (e.g. Rasconi et al. 2009, Taube et al. 2019). Yet, it is generally assumed that the parasites play not only an important role in their host population dynamics causing a significant impact on the planet’s carbon cycle (e.g. by terminating algal blooms; Gleason et al. 2015; Frenken et al. 2016; Jephcott et al. 2016) but constitute also a highly nutritional food source for zooplankton (Agha et al. 2016; Frenken et al. 2017). Being rich in fatty acids and sterols (Gerphagnon et al. 2018), which are absent for instance in cyanobacteria, parasitic chytrids may create trophic links between low-quality phytoplankton and zooplankton (so-called ‘mycoloop’; Kagami et al. 2014). Also, chytrid infection can boost carbon availability when large inedible diatoms or poorly edible filamentous cyanobacteria dominate phytoplankton communities (e.g. Kagami et al. 2007; Frenken et al. 2020).

#### *Batrachochytrium* as the cause of amphibian population declines

Frogs and salamander populations in many parts of the world are rapidly disappearing. Infection of amphibian hosts by fungal pathogens in the order *Chytridiales*, *Batrachochytrium dendrobatidis* (denoted Bd) and *B. salamandrivorans* (Bsal), has been implicated in many population declines. Upon infecting the skin of susceptible amphibians, these chytrids can cause clinical signs of the disease chytridiomycosis. In some species, infected individuals face near certain death. In others, sublethal effects of infection may result in life-history changes that reduce population growth. Over the past 50 years, population declines in more than 500 species and extinctions of at least 90 species have been attributed to chytridiomycosis (Scheele et al. 2019; but see Lambert et al. 2020).

The disease was first recognized in the late 1990s. Zoopathologists were puzzled by deaths in captive colonies of several amphibian species. Histological examination revealed organisms in the skin of preserved frogs and toads that were associated with severe skin dermatitis. The organisms initially were identified as fungal-like protists (Nichols et al. 1996). Similar structures in the skin of dying Australian frogs were described as *Perkinsus*-like protists (L. Berger, pers. comm.). Only when Nichols consulted Joyce Longcore was the error recognized. First from electron micrographs and then upon culturing the fungus, Longcore recognized taxonomically distinctive features of the pathogen that allowed her to identify it as a member of the *Chytridiales*. The development of the thallus and the ultrastructure of the zoospores differed sufficiently from those of other chytridialean genera that *B. dendrobatidis* was described as a new species in a new genus (Longcore et al. 1999). Later, from dying fire salamanders (*Salamandra salamandra*) in The Netherlands, Martel et al. (2013) isolated another chytrid pathogen, *B. salamandrivorans* (Bsal).

**Fig. 9:**
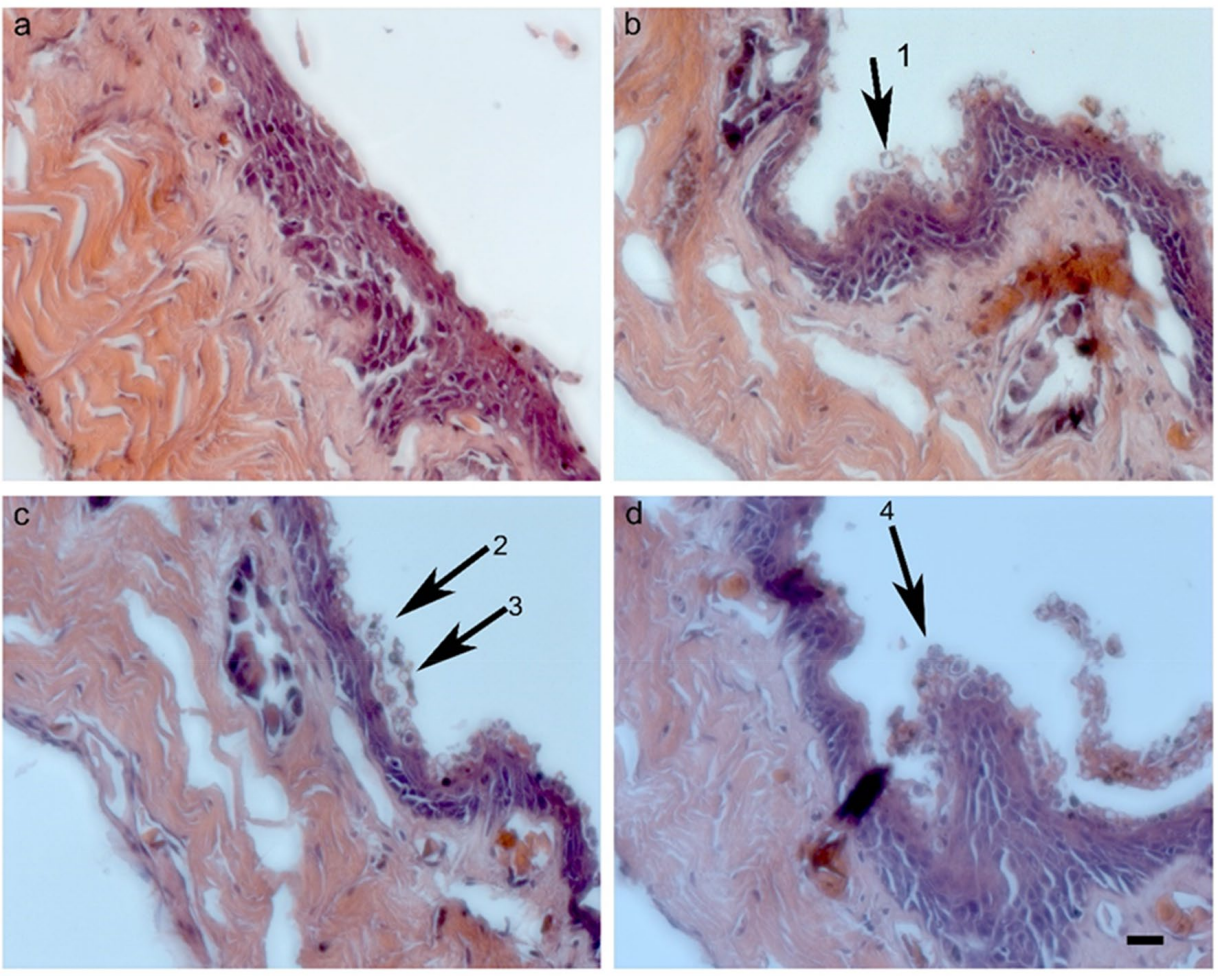
Skin histology of infected *Litoria caerulea*. Histological sections from BdAsia-1 treated groups. **a** Less infected skin region, note thickened epidermis. **b**–**d** Severe skin infection in individuals with high Bd infection loads. 1 a nearly empty zoosporangium. 2 a mature zoosporangium with zoospores in sloughing skin. 3 an empty zoosporangium that already has released zoospores in sloughed skin. 4 a mature zoosporangium with eight zoospores, about to be released. All four images are at the same magnification. Scale bar = 20 μm. Adapted from Fu and Waldman (2019)

Bd and Bsal have motile, asexual zoospores with a single flagellum. Infection begins as zoospores infiltrate and colonize the superficial layers of hosts’ skin or other keratinized tissues such as larval mouthparts. Zoospores minimally require aqueous films to disperse, but infection can occur even in terrestrial species as they require moist microhabitats. Once in contact with the epidermis, zoospores inhabit the skin and develop a germ tube, which grows through epidermal layers (Fig. 9). The germ tube swells to produce a thallus and smooth-walled zoosporangia (Berger et al. 2005). When the zoosporangia mature, new zoospores are released through discharge papillae that project out of sloughing skin into the environment, continuing the cycle of infection. Bd typically causes hyperplasia and hyperkeratosis of superficial epidermal layers, whereas Bsal infection often is associated with superficial epidermal erosion and deeper ulcerations (Martel et al. 2013).

Curiously, amphibian population declines and species extinctions attributed to Bd were found in Central America and Australia around the same time (Berger et al. 1998). The puzzle of how Bd could simultaneously emerge as a pathogen on opposite sides of the world remains unsolved. Nonetheless, shortly after these epizootics were reported, Bd was identified in dying frogs in New Zealand (Waldman et al. 2001), North America (Bradley et al. 2002), and Europe (Garner et al. 2005). Bd now has been found on every continent except Antarctica, and has been associated with population declines or extirpations everywhere except Asia.

Where the fungus became endemic, some amphibian hosts have evolved effective defenses against it including secretion of antimicrobial peptides (Rollins-Smith et al. 2005; Woodhams et al. 2007), adaptive immune responses (Rollins-Smith 2020; Zamudio et al. 2020), and commensal bacteria that mitigate fungal effects (Harris et al., 2006). Bd has been associated mostly with declines in anurans, while Bsal’s effects so far seem limited to urodeles (Farrer 2019). Coinfection of some salamanders by Bd and Bsal suggests that the pathogens may act synergistically to increase morbidity and mortality (McDonald et al. 2020). Acquired immunity may be possible, as pre-infection by less virulent Bd variants can confer resistance to more virulent ones in frogs and some salamanders (Greener et al. 2020).

Analyses of host transcription following infection may point to Bd and Bsal’s different modes of action (Farrer et al. 2017). The genome size of Bd (23.7 Mb) is smaller than that of Bsal (32.6 Mb). Bsal contains three times as many genes in the M36 metalloprotease family and CBM18 genes (cell-surface proteins) that are thought to degrade host tissue and contribute to pathogenicity (Farrer et al. 2013, 2017; Joneson et al. 2011). When Bd infects hosts, large transcriptional responses ensue, including both up-regulated innate and adaptive immunity genes and down-regulated mucin genes. Salamanders, however, fail to show similar responses to Bsal infection (Farrer et al. 2017).

#### Diagnosis of chytridiomycosis

Initial surveys for infection by Bd required laborious histological methods, and owing to poor preservation of specimens, diagnoses based on necropsies sometimes generated less than certain results. Other fungi (e.g. *Basidiobolus ranarum*) were identified in amphibian skin that appeared to cause similar superficial skin infections (Taylor et al. 1999). Unambiguous identification of Bd infection was facilitated by staining with polyclonal antibodies (Berger et al. 2002). Soon thereafter, molecular identification of Bd was made possible by real-time polymerase chain reaction (PCR) (Boyle et al. 2004) and nested PCR protocols (Goka et al. 2009). For Bsal diagnosis, real-time PCR protocols also have been established (Blooi et al. 2013). Today, virtually all surveys for Bd and Bsal are based on PCR assays of skin swabs. Surveys of infection prevalence can be more expeditiously conducted using these methods, but subjects with low chytrid loads sometimes are erroneously inferred to be free of infection (Shin et al. 2014). PCR techniques have been refined to permit retrospective studies of infection in museum specimens, revealing that amphibians may have been infected by Bd in many parts of the world over the past 100 years or longer (Goka et al. 2009; Rodriguez et al. 2014; Fong et al. 2015; Talley et al. 2015).

#### Asian origin

The simultaneous emergence of chytridiomycosis as an infectious disease around the world might suggest that the virulence of amphibian chytrid pathogens, already geographically widely distributed and colonizing amphibian skin, was triggered by global environmental changes. Alternatively, hosts may have evolved resistance to, or tolerance of, endemic chytrid lineages. Then, as novel variants of the pathogens spread, facilitated by growth in the international amphibian trade, hosts were unable to respond efficaciously to them. Recent molecular evidence favors the latter hypothesis.

Early genetic studies, based on multilocus sequencing, failed to find significant variation among Bd isolates from African, Australian, Panamanian, and North American host species. This suggested that the pathogen represented a recently emerged clone (Morehouse et al. 2003). However, further studies revealed substantial genetic variation among Bd lineages, some endemic to particular geographic regions where effects on hosts were not apparent. Disease outbreaks followed by population extirpations and species extinctions more often occurred when host species were naïve to Bd or became infected by novel variants.

The highest diversity of Bd variants has been found on the Korean peninsula (Bataille et al. 2013), presumably resulting from the pathogen’s long evolutionary history there. Full genome sequencing of Bd isolated from fire-bellied toads (*Bombina orientalis*) supports this hypothesis (O’Hanlon et al. 2018). Moreover, the endemic lineage BdAsia-1 infecting Korean amphibians appears to be in mutation-drift equilibrium, as expected of long established host–pathogen interactions (O’Hanlon et al. 2018). Phylogenetic analysis reveals that BdAsia-1 is basal to other Bd lineages and overlaps extensively with global Bd lineages. These findings suggest that Bd originated in Asia and then radiated around the world between 10,000 and 40,000 years ago (Rosenblum et al. 2013). Byrne et al. (2019) found another basal lineage, BdAsia-3, in Southeast Asia using a custom amplicon sequencing assay they developed that genotypes assorted regions of the Bd genome. Other potential basal lineages may remain to be discovered in Asia. Bsal also appears widespread in Asia, having little if any effect on sympatric hosts, again suggesting an Asian origin (Laking et al. 2017; Fisher and Garner 2020).

Six major Bd lineages have been identified (Byrne et al. 2019; O’Hanlon et al. 2018) (Table 3). Initial focus was on areas where population dieoffs occurred. A “global pandemic Bd lineage” (BdGPL) is associated with most epizootics in Australia, Europe and the Americas (Byrne et al. 2019; O’Hanlon et al. 2018). The hypervirulence of this lineage initially was hypothesized to arise from a hybridization event (Farrer et al. 2011) but this no longer seems likely (Fisher and Garner 2020). BdGPL is the most recent derived lineage, and dominates in most parts of the world except Asia and eastern Africa (O’Hanlon et al. 2018).

**Table 3:** Worldwide distribution of amphibian chytrid fungi *Batrachochytrium dendrobatidis* (Bd) and *B*. *salamandrivorans* (Bsal)

Lineage	Asia	Africa	Europe	Americas	Oceania
BdAsia-1/BdCH	**●**		**○**		
BdAsia-2/BdBrazil	**○**			**○**	
BdAsia-3	**●**				
BdCape		**●**	**○**	**●**	
BdGPL	**○**	**○**	**○**	**●**	**●**
Bsal	**○**		**○**		

For Bd, filled circles denote dominant lineages for each continent (O’Hanlon et al. 2018; Byrne et al. 2019)

BdCape was isolated from frogs in South Africa and was subsequently identified in other parts of Africa as well as Great Britain and Central America (Farrer et al. 2011; O’Hanlon et al. 2018). *Xenopus* frogs, which were routinely used for human pregnancy tests beginning in the 1930s, were thought to have spread Bd around the world (Weldon et al. 2004), but genetic evidence is not consistent with this hypothesis. BdCH initially was isolated from the midwife toad (*Alytes obstetricans*) in Switzerland (Farrer et al. 2011) but clusters closely with BdAsia-1 (O’Hanlon et al. 2018).

American bullfrogs (*Rana catesbeiana*) farmed on several continents, are tolerant of Bd so may spread the pathogen to native species where they are introduced. In Korea, infection loads of *R. catesbeiana* are higher than those of native species, and they appear to carry principally the lineage BdAsia-2 (Bataille et al. 2013; O’Hanlon et al. 2018). Similarly, in the USA and Brazil, *R. catesbeiana* serves as a reservoir of BdBrazil, which apparently has spread to native species (Schloegel et al. 2012). BdBrazil, also found on frogs in Japan, clusters with BdAsia-2 into one distinct lineage (O’Hanlon et al. 2018). Genetic sequencing thus provides supportive evidence that trade in bullfrogs played a significant role in spreading Bd around the world before reestablishing itself in Asia (Fisher and Garner 2020).

In addition to the six major lineages, recombinants or hybrids of BdAsia-2/Brazil and BdGPL, as well as other undetermined lineages, have been found (Byrne et al. 2019; Fisher and Garner 2020). Sexual recombination in Bd was first reported via internal transcribed spacer (ITS) region sequencing (Schloegel et al. 2012) and was later supported by whole genome sequencing (O’Hanlon et al. 2018). Recombination between lineages may produce high virulence, as evidenced by studies of a BdBrazil/BdGPL cross (Greenspan et al. 2018).

Bd lineages vary not only in genomic information but also in virulence and morphology. For example, BdCH has larger sporangia than BdCape or BdGPL (Farrer et al. 2011). BdBrazil zoospores are smaller than those of BdGPL (Becker et al. 2017). In addition, zoospore and zoosporangium characteristics vary even among nearby, genetically connected populations, suggesting local host adaptation or phenotypic plasticity (Fisher et al. 2009; Farrer et al. 2011; Lambertini et al. 2016). As BdAsia-1 appears hypervirulent to susceptible species outside Asia (Fu and Waldman 2019), further characterization of this basal lineage is necessary to delineate the association between virulence and morphology (Fisher et al. 2009).

### Taxonomy of nephridiophagids

#### The fungal kingdom adopts the obligate insect-pathogenic nephridiophagids (Nephridiophagidae): *Nephridiophagales*

**Fig. 10:**
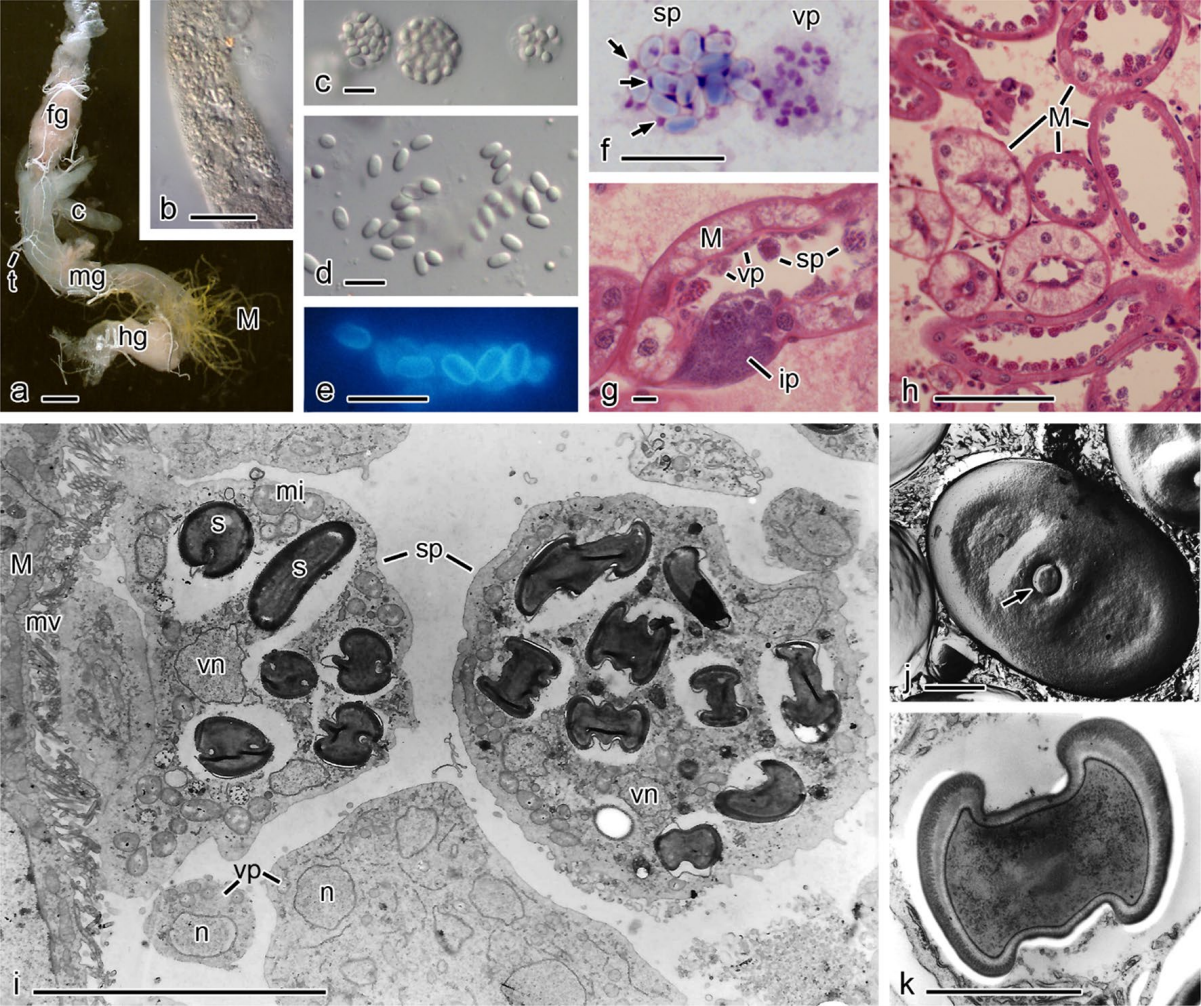
Life cycle stages of *Nephridiophaga blattellae* (*Chytridiomycota*). **a** Extracted gut of *Blattella germanica* (German cockroach) with Malpighian tubules (M) at the border of midgut (mg) and hindgut (hg) as habitat for *Nephridiophaga blattellae*. c, caeca; fg, foregut; t, trachea. **b** Infected Malpighian tubule full of parasite stages. Differential inferference contrast (DIC). **c** Three sporogenic plasmodia of different sizes. DIC. **d** Spores released from ruptured sporogenic plasmodia. DIC. **f** Giemsa stained smear of infected Malpighian tubules. Residual vegetative nuclei (arrows) in sporogenic plasmodium (vp); vegetative plasmodium (vp) with numerous nuclei. **e** Spores labelled with the fluorescent stain Calcofluor White reveal the presence of chitin in the spore walls. **g**, **h** Paraffin sections of *B. germanica* stained wirh hemalaun eosin. Rarely, intracellular plasmodia (ip) are found in epithel cells of the Malpighian tubules. Mostly, vegetative and sporogenic plasmodia develop in the lumen of the tubules, generally attached to the epithelium. **i** Ultra-thin section of Malpighian tubule. The plasmodia of *Nephriophaga* attach to the microvilli border (mv) or are free in the lumen. Sporogenic plasmodia with mature spores (s) and residual vegetative nuclei (vn) in the mother cell cytoplasm. Vegetative plasmodia with nuclei (n). mi, mitochondria. **j** Freeze-etch sample of a mature spore shows a little central cap at the spore opening (arrow). Ultrathin cross-section of mature spore with thick spore wall at the border and thin spore wall at the flat upper and lower sides. Scale bars: **a** = 1 mm, **b** = 50 µm, **c**–**g**, **i** = 10 µm, **h** = 100 µm, **j**, **k** = 1 µm

The nephridiophagids are unicellular obligate parasites that infect the Malpighian tubules of arthropods where especially in the lumen different life cycle stages can be densely packed (Fig. 10). Their life cycle includes a vegetative phase with multinucleate plasmodia that divide into oligo- and uninucleate cells, and a generative phase, in which the vegetative plasmodia transform into sporogenic plasmodia that form uninucleate spores. Mature spores with a thick chitinous wall are released with the host feces facilitating infection of further individuals by oral uptake (e.g. Woolever 1966; Radek et al. 2002). So far, nephridiophagids have been found mostly in cockroaches and beetles (Radek and Herth 1999) but are also known from other, more distantly related hosts such as the European earwig *Forficula auricularia* (Ormières and Manier 1973) or the honey bee *Apis mellifera* (formal description of the genus *Nephridiophaga*; Ivanić 1937). Although generally assumed to coevolve with their hosts, transmission of nephridiophagids between only distantly related cockroaches has recently been documented in a study, in which, however, the different cockroach species were kept together in the same cultural area (Strassert et al. 2021).

Being poor in morphological characteristics, the phylogenetic affiliation of nephridiophagids remained controversial for many decades (e.g. Perrin 1906; Ivanić 1937; Sprague 1970; Purrini and Rohde 1988; Lange 1993). Based on molecular phylogenetic analyses of the SSU rRNA gene of the nephridiophagid *Nephridiophaga blattellae* (from the German cockroach *Blattella germanica*), an assignment to the fungi was first been proposed by Wylezich et al. (2004). The fungal nature of nephridiophagids was then later confirmed with statistical support by adding the SSU rRNA gene sequences of two further *Nephridiophaga* species to the phylogenetic analysis (Radek et al. 2017). Most recently, molecular tree inferences from a concatenated alignment of SSU and LSU rRNA genes of nine nephridiophagid species have finally uncovered their robust assignment to the phylum *Chytridiomycota* (Strassert et al. 2021). Whereas these analyses showed a clear distinction of nephridiophagids from other order-level clades within the *Chytridiomycota*, confirming the order *Nephridiophagales*, their closer relationship to one of these clades remains uncertain (a sister relationship to the *Cladochytriales* is hypothesized; Strassert et al. 2021).

#### *Nephridiophagales* Doweld 2014 emend. Strassert and Radek 2021

Thalli reduced to reproductive structures comprising sporogenic plasmodia; filamentous and flagellated stages absent or not observed; Spores thick-walled, flattened, oval to elongate, uninucleate or binucleate, resembling spore sori; vegetative and sporogenic life cycles stages generally develop in the lumen of Malpighian tubules and rarely intracellular in their epithelium; in extremely high infections of *Blattella germanica*, stages also occurred in the fat bodies and the male accessory glands; obligate parasitic/commensal or mutualistic in arthropods.

Genus: *Nephridiophaga* Ivanić 1937

Existing type species: *N. apis* Ivanić 1937

Proposed type species: *N. blattellae* (Crawley 1905; Woolever 1966; Fig. 10).

Habitat: Malpighian tubules of *Blattella germanica* (Fig. 10a, b, h). Sporogenic plasmodia contain 10–30 spores and residual vegetative nuclei (Fig. 10c, f, i). Native mature spores are oval, measuring 5.5 (5.0–6.0) × 3.2 (2.5–3.5) µm, and are flattened (about 2.5 µm thick) (Fig. 10d, j, k). Central spore opening on flat side (Fig. 10j); chitin-containing cell wall (Fig. 10e). Intra- and extracellular, multinucleate vegetative plasmodia of different sizes (e.g. 20 µm) (Fig. 10f, g–i).

The illustration of *N. apis* by Ivanić (1937) can be interpreted as the holotype but an epitype should be ‘justifiable’, e.g., from the same host (as these organisms are generally host specific). However, *N. apis* was never re-isolated from honey bees. Although Plischuk and Lange (2011) reported light microscopic stages of *N. apis* from Argentinian honey bees, we could not prove *Nephridiophaga* by transmission electron microscopy or molecular analysis in a sample provided (but only microsporidia). If the relatedness of *N. apis* to the other species of *Nephridiophaga* is doubtful, all the other species of *Nephridiophaga* would require a new generic name and published combinations. This would be highly destabilizing and thus the only option to maintain stability in the naming of the majority of these species is a conserved type. The first and most numerous descriptions of nephridiophagids are from cockroaches (Lutz and Splendore 1903; Crawley 1905; Wijayawardene et al. 2020). Therefore, and because *N. blattellae* (Fig. 10) is the best investigated species of the whole group (Crawley 1905; Léger 1909; Woolever 1966; Radek and Herth 1999; Radek et al. 2017; Strassert et al. 2021), we propose this species as the new type species.

## Short history of proven nephridiophagids and given genus names

The genus *Nephridiophaga* was introduced for *N. apis* from the honey bee *Apis mellifera* by Ivanić (1937). The name refers to the detrimental effect of an infection to the Malpighian tubules (‘feeders of nephridia’). However, nephridiophagids have been described earlier under different genus names, mainly because their affiliation to known groups of spore-forming unicellular pathogens was unknown. The first report was by Lutz and Splendore (1903) for nephridiophagids from the American cockroach *Periplaneta americana*. The authors interpreted the stages as microsporidia and thus described them as *Plistophora periplanetae*. In 1905, Crawley found a similar infection in the German cockroach *Blattella germanica*. Since he believed in a haplosporidian nature of the parasite, he named it *Coelosporidium blattellae*. In 1909, Léger created a new genus *Peltomyces* (referring to mycetozoans) for new nephridiophagids from the beetle *Olocrates hyalinus* and the earwig *Forficula auricularia*. Morphology and life cycle stages of the two new species closely resemble that of *Nephridiophaga* and this later led to a synonymization with the genus *Nephridiophaga* (Woolever 1966; Radek and Herth 1999). Molecular data would be necessary to justify the recognition of the genus *Peltomyces*. In the following years, an intense discussion was held concerning the true affiliation of the nephridiophagids, the genus and family names they should be given, and which spore formers really belong to this group (e.g. Sprague 1970; Toguebaye et al. 1986; Woolever 1966; Purrini and Weiser 1990; Lange 1993; Radek and Herth 1999). In addition to the genus *Nephridiophaga*, two further (monotypic) genera most probably represent nephridiophagids: *Coleospora* (*binucleata*) from the beetle *Gonocephalum arenarium* (Gibbs 1955), and *Oryctospora* (*alata*) from the beetle *Oryctes monoceros* (Purrini and Weiser 1990). These genera differ morphologically from *Nephridiophaga* either by possessing elongate mature spores with two nuclei (*Coleospora*), or by having spores with lateral protrusions and a polar opening.

Other species of *Nephridiophaga*:

*N. archimandrita* R. Radek, Wellmanns, A. Wolf 2011

*N. blaberi* Fabel, (Radek et al. 2002)

*N. blattellae* (H. Crawley) P. Woolever 1966

*N. forficulae* (Léger 1909) Ormières & Manier 1973

*N. javanicae* J.F.H. Strassert & R. (Strassert et al. 2021)

*N. meloidorum* (Purrini & Rhode 1988) Lange 1993

*N. lucihormetica* R. Radek, Wellmanns, A. Wolf 2011

*N. maderae* R. Radek, Owerfeldt, Gisder & Wurzbacher 2017

*N. ormieresi* (Toguebaye et al. 1986) Purrini & Weiser 1990

*N. periplanetae* (Lutz and Splendore 1903) Lange 1993

*N. postici* J.F.H. Strassert & (Strassert et al. 2021)

*N. schalleri* (Purrini & Rhode 1988) Lange 1993

*N. tangae* (Purrini et al. 1988) Lange 1993

*N. xenoboli* P.N. Ganapati & C.C. Narasimhamurti 1960

For the reason of a diverging spore type (spherical with two valves) *N. xenoboli* probably has to be removed from the genus *Nephridiophaga* according to Radek and Herth (1999).

## Genomes and the rise of phylogenomic studies

**Fig. 11:**
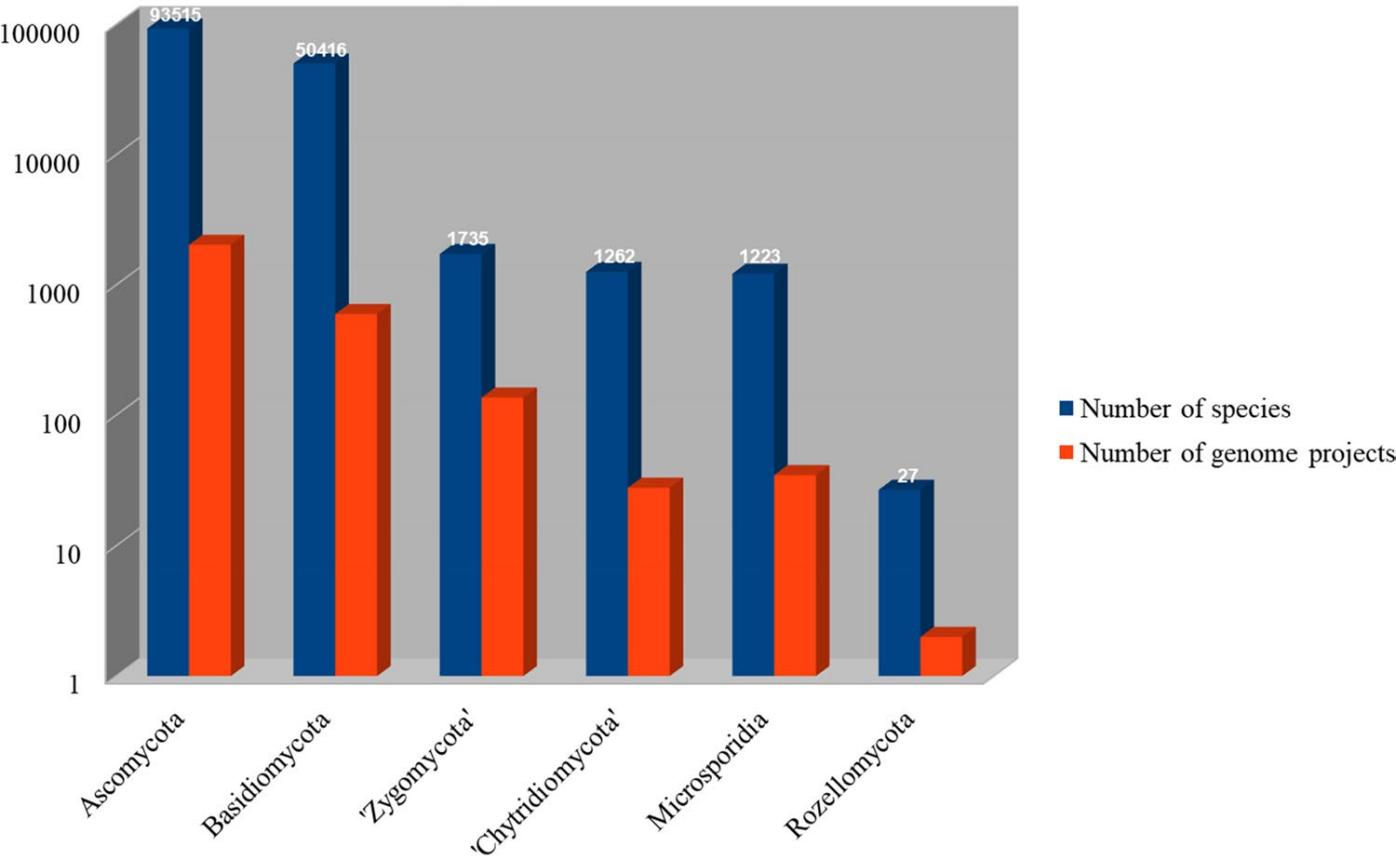
Species counts *versus* genome counts among the most predominant fungal phyla (accessed at SpeciesFungorum and GenBank, respectively, as of 19th April, 2021). The terms ‘*Chytridiomycota*’ and ‘*Zygomycota*’ were used in a colloquial sense to depict the basal lineages of zoosporic fungi (*Chytridiomycota and Blastocladiomycota*) and zygosporic fungi (*Entomophthoromycotina, Glomeromycotina, Kickxellomycotina, Mortierellomycotina, Mucoromycotina, Zoopagomycotina*)

Currently, a total of 2845 fungal genomes are available (GenBank, accessed on 19th April, 2021). The species-genome ratio among basal fungi is comparable to that of the derived Dikarya meaning a catch up for genome projects among basal fungi which was highly encouraged by Shelest and Voigt (2014). However, a lack of genome projects of the missing link and parasitic taxa still hampers phylogenomic analyses with sufficient statistical clade stability supports. We can conclude that an acceleration of both a search for missing link species in rarely sampled habitats and an increase of fungal genome projects of evolutionary link taxa will be beneficial for the phylogenetic reconstruction of basal fungi (Fig. 11).

## Conclusions and prospectives

This paper provides an overview of current species concepts, ecology, phylogeny, and the distribution of basal fungal lineages. We summarize existing knowledge on the phylogeny and review the taxonomy of selected groups. The diversity and ecology of basal fungal lineages is obscure but new groups are being described at a fast rate. We highlight the impact of two chytrid species belonging to *Chytridiomycota* on amphibian populations. It is increasingly important to know the roles of more basal fungi in global ecosystems. Discovery of new taxa has led to the dramatic changes in the phylogeny and diversity of fungi in basal clades, but the taxonomy is still debated. Additional taxonomic studies of diverse genera in the basal lineages are required in the future.
